# Facile carrier-assisted targeted mass spectrometric approach for proteomic analysis of low numbers of mammalian cells

**DOI:** 10.1038/s42003-018-0107-6

**Published:** 2018-08-06

**Authors:** Tujin Shi, Matthew J. Gaffrey, Thomas L. Fillmore, Carrie D. Nicora, Lian Yi, Pengfei Zhang, Anil K. Shukla, H. Steven Wiley, Karin D. Rodland, Tao Liu, Richard D. Smith, Wei-Jun Qian

**Affiliations:** 1 Biological Sciences Division, Pacific Northwest National Laboratory, Richland, 99354 WA USA; 2 Environmental Molecular Sciences Laboratory, Pacific Northwest National Laboratory, Richland, 99354 WA USA; 3 Key Laboratory of Cancer Proteomics of Chinese Ministry of Health, Xiangya Hospital, Central South University, Changsha, 410008 Hunan People’s Republic of China

## Abstract

There is an unmet technical challenge for mass spectrometry (MS)-based proteomic analysis of single mammalian cells. Quantitative proteomic analysis of single cells has been previously achieved by antibody-based immunoassays but is limited by the availability of high-quality antibodies. Herein we report a facile targeted MS-based proteomics method, termed cPRISM-SRM (carrier-assisted high-pressure, high-resolution separations with intelligent selection and multiplexing coupled to selected reaction monitoring), for reliable analysis of low numbers of mammalian cells. The method capitalizes on using “carrier protein” to assist processing of low numbers of cells with minimal loss, high-resolution PRISM separation for target peptide enrichment, and sensitive SRM for protein quantification. We have demonstrated that cPRISM-SRM has sufficient sensitivity to quantify proteins expressed at ≥200,000 copies per cell at the single-cell level and ≥3000 copies per cell in 100 mammalian cells. We envision that with further improvement cPRISM-SRM has the potential to move toward targeted MS-based single-cell proteomics.

## Abstract (web summary)

Tujin Shi et al. report a mass spectrometry-based proteomics approach, cPRISM-SRM, that allows for accurate quantification of proteins in small numbers of mammalian cells through the use of a carrier protein to prevent sample loss. The sensitivity of cPRISM-SRM allows for measurement of the 2500 most abundant proteins in a human cell.

## Introduction

Recent advances in nucleic acid sequencing technologies allow for precise measurement of the transcriptome in single cells at a comprehensive genomic scale^1,2^. However, single-cell proteomics technologies are lagging far behind, but are equally important to genomics technologies^3–7^. Currently, single-cell proteomics measurements exclusively rely on antibody-based immunoassays for targeted proteomic analysis of single cells^5,8^. However, they have inherent limitations (e.g., low multiplex and enormous challenges of generating high-specificity antibodies, especially for protein mutations and posttranslational modifications). They also generally lack quantitation accuracy to estimate absolute protein amount or concentration^8,9^. Mass spectrometry (MS)-based targeted proteomics is a highly attractive alternative or complementary to antibody-based assays for single-cell proteomics analysis because it is antibody-free as well as its inherent high multiplexing capability, specificity, and quantitation precision and accuracy^10^. With recent advances in separations and MS instrumentation, the most sensitive MS platform can detect peptides at ~10–100 zmol (i.e., 6000–60,000 molecules) for sub-nanogram amounts of peptide mixtures from bulk cell digests^11–17^. In theory, such sensitivity is sufficient to quantify ~25–55% of the whole proteome of a single mammalian cell (i.e., ~4000–8500 proteins out of ~15,000 proteins in a single HeLa cell)^18^ assuming 100% sample recovery during sample processing and high-efficiency ion generation and transmission to MS. However, there is an unmet technical challenge in sample preparation for effectively lossless processing of single mammalian cells for MS analysis.

Single-cell MS was recently reported for proteomic analysis of very large single cells^19–24^, such as oocytes with ~100–1000 µm in diameter and ~0.1–100 µg of proteins per cell^25^. However, it remains challenging to apply current MS platforms to single mammalian cells because most are ~10–100-fold smaller in diameter with ~10^3^–10^6^-fold less protein content (i.e., ~10 µm in diameter and ~100 pg per cell) than oocytes or early stage embryo cells^25^. Progress in mass-limited sample processing (e.g., single-tube preparation or nanoPOTS and online processing system)^26,27^ has been recently reported for enabling effective processing of hundreds and thousands of mammalian cells (i.e., 10–1000 ng of total protein amount) with identification of ~1000–3000^16,27^ and ~3000–4000 proteins^12,21,28–30^, respectively. Nevertheless, when sample size becomes smaller (close to single cells), there is increasingly substantial and unavoidable loss through contact-surface adsorption regardless of current sample preparation methods^28,31^.

To address this issue we developed a facile targeted mass spectrometric approach, termed cPRISM-SRM (carrier-assisted high-pressure, high-resolution separations with intelligent selection and multiplexing coupled to selected reaction monitoring), for enabling proteomic analysis of very low numbers of mammalian cells. cPRISM-SRM capitalizes on the use of excessive exogenous protein as a carrier to minimize sample loss together with our recently developed high-resolution PRISM^32^ method to reduce the wide dynamic range of protein concentrations caused by the addition of protein carrier. cPRISM-SRM uses a sensitive-targeted MS platform (e.g., SRM)^10,33^ for proteomic analysis of few cells. We used human mammary epithelial cells (HMEC) as a model system because they are highly representative of most mammalian cells, with a wide dynamic concentration range, and we have extensively characterized its proteome and protein abundance profile^34–37^. We have shown that cPRISM-SRM enables detection of high- to moderate-abundance proteins in single HMEC cell equivalents and low-abundance proteins in ~100 HMEC cell equivalents, ~3–4 orders of magnitude lower than the cell number required for current targeted MS methods (typically ~10^5^–10^6^ cells^32,37^).

## Results

### cPRISM-SRM performance in HMEC cell equivalents

**Fig. 1:**
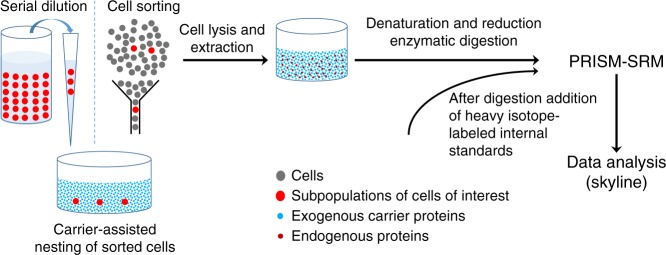
Schematic diagram of the cPRISM-SRM workflow. A small number of cells are isolated either by serial dilution or cell sorting and collected into a container with large amounts (~50 µg) of carrier proteins to prevent undesired sample loss. Commonly used digestion protocols are used for sample processing to generate tryptic peptides without any further modification. After digestion and sample cleanup heavy isotope-labeled internal standards are added to the peptide mixtures. Highly sensitive PRISM-SRM is then used for precise quantification of surrogate peptides from proteins of interest with reducing the significantly increased dynamic concentration range caused by the addition of carrier proteins. The freely-available open-source Skyline software is used for SRM data analysis

The development of cPRISM-SRM was inspired by our observation of reliable MS detection of extremely low-abundance proteins through extensive fractionation, apparently because high-abundance proteins have served as an effective carrier to prevent their loss^37^. Thus, we reasoned that the addition of exogenous carrier proteins could prevent protein loss from single or few cells during sample processing, and the use of extensive fractionation (e.g., PRISM) could reduce the increased dynamic concentration range for sensitive SRM detection. Figure 1 depicts the workflow of cPRISM-SRM, where isolated small numbers of cells are collected into a container preloaded with sufficient amounts of carrier protein. BSA was selected as the carrier protein because of its well-established utility for preventing protein or peptide loss. BSA is readily available in large quantities of for the entire method development with high purity (>99%) and negligible contamination (Supplementary Data 1, 2). In addition, BSA displays broad distribution of its tryptic peptides (i.e., different hydrophobicity) across the entire liquid chromatography (LC) separation window (Supplementary Fig. 1 and Supplementary Data 3). However, the addition of the BSA carrier results in an increase of dynamic range in protein concentration by ≥4 orders of magnitude (e.g., mixing ten mammalian cells, equivalent to ~1 ng in protein mass, with typical ~50 µg of BSA carrier). To compensate for this, our recently developed high-resolution PRISM^32^ was used to selectively enrich target peptides and remove potential co-eluting interferences **(**Fig. 1 and Supplementary Fig. 2**)**. Following PRISM, peptide fractions of interest are directly subjected to LC-SRM analysis for protein quantification.

The sensitivity and precision of cPRISM-SRM were initially evaluated by targeted quantification of several high- and moderate-abundance EGFR pathway proteins using small numbers of HMEC cell equivalents taken from bulk HMEC digests (Supplementary Data 4–7 and Supplementary Table 1). We chose to target EGFR pathway proteins due to the availability of heavy peptide internal standards with established SRM assays (Supplementary Data 4) and established protein copies per cell from our recent study (Supplementary Data 6)^37^. Another reason we chose this pathway is because EGFR pathway is one of the most important signaling pathways in cancer^38,39^. We used HMEC cell equivalents to eliminate any biological variation due to stochastic sampling of small numbers of intact cells that might complicate the evaluation of cPRISM-SRM analytical performance.

Different numbers of cells from bulk HMEC digests (equivalent to 1–1000 cells) were spiked into ~25 µg of BSA digests to mimic intact HMEC nested into excessive amounts of carrier protein. Following PRISM fractionation, a given fraction of interest was selected and analyzed by LC-SRM. The obtained limits of detection (LODs) and limits of quantification (LOQs) were used to evaluate the detection sensitivity. The peptide recovery throughout the cPRISM-SRM workflow was evaluated by comparison of the SRM signal of heavy peptide standards between cPRISM-SRM and carrier-assisted LC-SRM (i.e., cLC-SRM) for measuring the same equivalent number of cells with the same amount of spiked-in heavy standards. The precision was assessed by correlation analysis of the SRM signal ratio (L/H) of endogenous light peptides over heavy standards between the small number of cells by cPRISM-SRM and bulk cells by LC-SRM.

**Fig. 2:**
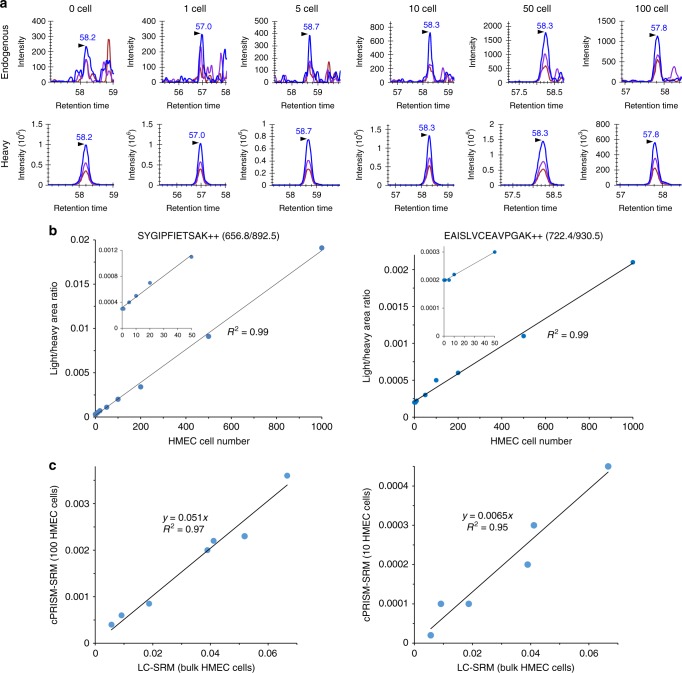
Sensitivity and accuracy of cPRISM-SRM assays in HMEC cell equivalents (i.e., small equivalent numbers of HMEC cells taken from bulk cell digest). **a** Extracted ion chromatograms (XICs) of transitions monitored for SFEDIHHYR derived from K/NRAS at different equivalent numbers of HMEC cells. 656.8/892.5 (*blue*), 656.8/308.1 (*purple*), 656.8/421.2 (*chestnut*). **b** Calibration curves for quantifying K/NRAS and SCH1 with the use of the best interference-free transition. Inset plots show the details of the low number of cell equivalents. **c** Correlation curves of the L/H ratio between 100 or 10 HMEC cell equivalents from cPRISM-SRM and bulk HMEC cells from LC-SRM. In the ten HMEC cell equivalents, six surrogate peptides were detected by cPRISM-SRM, and subtraction of the background SRM signal was conducted due to major portion of endogenous SRM signal contributed by matrix background

Figure 2a depicts extracted ion chromatograms (XICs) of transitions monitored for SYGIPFIETSAK derived from K/NRAS at ~180,000 copies per HMEC cell. Clearly, cPRISM-SRM enabled us to detect endogenous SYGIPFIETSAK in a single HMEC cell equivalent with a signal-to-noise ratio (S/N) of 8 and ~300 zmol of detection sensitivity (Table 1 and Supplementary Table 2). Such level of sensitivity was further confirmed by the confident detection of multiple endogenous peptides from other EGFR pathway proteins present at low copy number in HMEC (e.g., EAISLVCEAVPGAK derived from moderate-abundance SHC1 at ~25,000 copies per cell in ten HMEC cell equivalents with the S/N ratio of 3 and ~400 zmol of detection sensitivity) (Supplementary Figs. 3–9 and Table 1). The resultant calibration curves have shown excellent linearity over a wide range of HMEC cell equivalents (Fig. 2b and Supplementary Figs. 3–9) with LOQs of 5–20 cells for high-abundance proteins and 10–100 cells for moderate-abundance proteins (Table 1).

**Table 1:** Limits of detection (LODs), limits of quantification (LOQs), and absolute sensitivity of cPRISM-SRM for selected surrogate peptides

Gene	Estimated protein copies per cell^a^	Surrogate peptide	Detection sensitivity			
			LOD (cell)	LOQ (cell)	LOD (zmol)	LOQ (zmol)
EGFR	354,000	LTQLGTFEDHFLSLQR	20	20	11,761	11,761
H/K/NRAS	213,232	LVVVGAGGVGK	5	5	1771	1771
K/NRAS	177,780	SYGIPFIETSAK	1	5	295	1477
NRAS	82,045	SFADINLYR	5	5	681	681
HRAS	68,452	SFEDIHQYR	50^b^	100^b^	5685	11,371
ADAM17	36,080	NIYLNSGLTSTK	1	100^c^	60	5993
SHC1	25,055	EAISLVCEAVPGAK	1	10	42	416

^a^Our recent study for absolute quantification of EGFR pathway proteins

^b^Significant matrix interference for endogenous peptide at 1–50 cell equivalents (Supplementary Fig. 7)

^c^Target peptide fractions at 20 and 50 cell equivalents were not located for LC-SRM analysis

The reproducibility of cPRISM-SRM was evaluated by analysis of processing replicates for two peptides SYGIPFIETSAK and LVVVGAGGVGK in 10 and 20 HMEC cell equivalents, respectively (Supplementary Fig. 10). The processing coefficients of variation (CoVs) for both peptides were below 15%, suggesting high reproducibility of cPRISM-SRM. No injection replicates for LC-SRM were performed for target peptides because the majority of the PRISM fraction samples (~16 µL out of the total ~20 µL) was used for one single LC-SRM analysis to maximize the detection sensitivity. Furthermore, the LC-SRM technical reproducibility has been well characterized with the CoV below 10%^10,32,40–43^. The peptide recovery throughout the cPRISM-SRM workflow was evaluated by comparison of the SRM signal of heavy peptide standards in small numbers of cell equivalents from cPRISM-SRM with that from cLC-SRM assuming ~100% recovery for cLC-SRM with direct injection. Our comparative analysis shows the overall peptide recovery of cPRISM-SRM ranging from 80 to 220% with the average recovery of 150% (Supplementary Fig. 11). Such high recovery was attributed to lower ion suppression in cPRISM-SRM than cLC-SRM due to high-resolution PRISM separation for nearly lossless target peptide enrichment while there is matrix effect for cLC-SRM analysis. Since the heavy standards essentially have the same physical and chemical properties as their corresponding light peptides, the endogenous light peptides in small numbers of cell equivalents should have the same peptide recovery as the heavy internal standards.

We next evaluated quantitation accuracy of cPRISM-SRM for small numbers of cell equivalents by head-to-head comparison with bulk cell measurements. Our correlation analysis indicated that there was a strong correlation (*R*^2^ > 0.95) in the L/H ratios of quantifiable peptides between 50 and 1000 HMEC cell equivalents by cPRISM-SRM and bulk HMEC digests by LC-SRM (Fig. 2c and Supplementary Figs. 12a–c), suggesting high quantitation accuracy of cPRISM-SRM for low numbers of cells. However, as the cell number decreased to ten, no significant correlation (*R*^2^ = 0.40) was observed (Supplementary Fig. 12d), primarily due to the major portion of SRM signal from the matrix background (i.e., the intercept on the *y*-axis) with different degree of contribution for each peptide (Supplementary Figs. 3–9). With the subtraction of the background SRM signal an excellent correlation was obtained with *R*^2^ = 0.95 between ten cell equivalents by cPRISM-SRM and bulk cell digests by LC-SRM (Fig. 2c). This result further demonstrates that cPRISM-SRM has high quantitation accuracy, and thus could be used for reliable protein quantification in a very small number of cells.

### Quantification of EGFR pathway proteins in intact HMEC cells

**Fig. 3:**
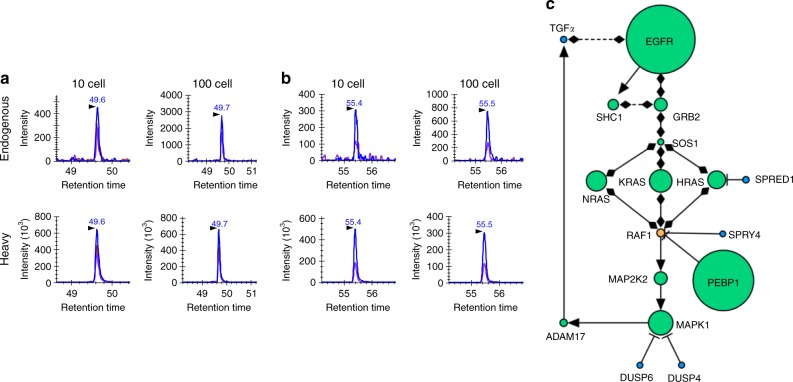
Comparison of SRM signal between 10 and 100 intact HMEC cells isolated from serial dilution measured by cPRISM-SRM: **a** XICs of transitions monitored for LVVVGAGGVGK derived from H/K/NRAS. 478.3/743.4 (*blue*), 478.3/644.4 (*purple*), 478.3/545.3 (*chestnut*). **b** XICs of transitions monitored for SFADINLYR derived from NRAS. 549.8/864.5 (*blue*), 549.8/793.4 (*purple*). **c** Quantification of EGFR pathway proteins in 100 intact HMEC cells by cPRISM-SRM. Detected proteins are in green, undetected proteins are in blue, unmeasured proteins are in orange. Activating interaction are shown as arrows, inhibiting interactions are shown as ‘T’ lines, and protein–protein interactions are shown as diamonds. Size of each node is directly proportional to protein abundance from our previous bulk HMEC measurement

With the demonstration of robustness in cell equivalents, we next applied cPRISM-SRM to measure EGFR pathway proteins in small numbers of intact human cells. Based on known protein abundance^37^ and the detection sensitivity of cPRISM-SRM, four surrogate peptides that represent high-abundance and moderate-abundance proteins, were comparatively measured in 10 and 100 intact HMEC cells from serial dilution of cell suspension, validated by optical microscopy, to evaluate its practical applicability (Fig. 3a, b and Supplementary Fig. 13). cPRISM-SRM enabled us to detect moderate-abundance SHC1 protein at ~25,000 copies per cell in ten intact HMEC cells with the S/N ratio of 5 (Supplementary Fig. 13c). When the cell number was increased to 100, the endogenous SRM signal of SHC1 peptide was increased accordingly with the S/N ratio of 8 (Supplementary Fig. 14). To elucidate the quantitation dynamic range of cPRISM-SRM, we measured EGFR pathway proteins with a wide range of protein abundance (~3000–350,000 copies per cell) in 100 HMEC cells (Fig. 3c). A majority of previously defined core EGFR pathway proteins^37^ were reliably detected and quantified by cPRISM-SRM, with the exception of extremely low-abundance negative feedback regulators (Fig. 3c and Supplementary Fig. 14). This result suggests that cPRISM-SRM can be used to study signaling pathways (e.g., revealing stoichiometric bottlenecks) in small numbers of cells. Significantly, the endogenous peptide from low-abundance SOS1, present at only ~3000 copies per cell, was detected in 100 HMEC cells with the S/N ratio of 4 (Supplementary Fig. 14), suggesting ~500 zmol of absolute sensitivity of cPRISM-SRM. This is consistent with the endogenous detection of SHC1 at ~25,000 copies per cell in ten HMEC cells with ~400 zmol of sensitivity. Thus, the detectability of endogenous peptides by cPRISM-SRM appears to be primarily determined by the total number of protein molecules (i.e., protein copies per cell × the total number of cells). Correlation analysis further confirmed quantitation accuracy for enabling analysis of small numbers of cells with correlation coefficient of *R*^2^ = 0.86 between 100 intact HMEC cells and bulk HMEC cells (Supplementary Fig. 15). This value was somewhat lower than that between 100 cell equivalents and bulk HMEC cells, presumably because the two sets of samples were processed at different batches. We further evaluated the reproducibility of the entire cPRISM-SRM pipeline, including sample processing, using a relatively large number of intact HMCE cells isolated by serial dilution (Supplementary Fig. 16). The overall processing CoVs for four replicates ranged from 8.5 to 23.6% with the average CoV of 13.4%, close to that of ~10% in PRISM-SRM^32,42^. This result suggests that cPRISM-SRM can provide reliable quantification for small numbers of intact mammalian cells.

## Discussion

We have shown that the integration of the carrier protein concept into our highly sensitive PRISM-SRM platform enables precise proteomic analysis of very low numbers of mammalian cells. cPRISM-SRM represents a significant advance toward targeted MS-based single-cell proteomics. For the overall analytical performance, cPRISM-SRM essentially has the same processing reproducibility, peptide recovery, and quantitation dynamic range as PRISM-SRM. The average processing CoV of small numbers of cells, including sample preparation, was below 15%, which is consistent with that of ~10% in PRISM-SRM^32,42^. When compared to cLC-SRM, high peptide recovery was observed with the average value of 150% (Supplementary Fig. 11), suggesting that the high-resolution PRISM fractionation resulted in greatly reduced co-eluting matrix interference and consequently lower ion suppression than cLC-SRM. Such high-resolution separation was also supported by another observation that the detectability of endogenous peptides in small numbers of cell equivalents was primarily determined by the total number of protein molecules. cPRISM-SRM was proved to achieve nearly full-baseline separation for BSA carrier-containing small numbers of cell equivalents because of ~200-fold improvement in sensitivity (i.e., the quantitation dynamic range of ~7 orders of magnitude) when compared to regular LC-SRM^32,36,37,42,44,45^. Because HMEC has typical size, protein composition, and a wide dynamic concentration range as most mammalian cells, cPRISM-SRM should be broadly applicable to low numbers of mammalian cells.

Using standard LC-MS instruments, cPRISM-SRM was demonstrated to enable detection and quantification of moderate-abundance proteins at ~25,000 copies per cell in 1 ng of cell lysate digests (equivalent to ten mammalian cells) and high-abundance proteins at ~200,000 copies per cell in 100 pg of cell lysate digests (equivalent to one single mammalian cell) (Supplementary Table 2). Such sensitivity is sufficient to detect the 2500 most abundant proteins in a single human cell^18^, which should be able to identify different states of cellular differentiation. Furthermore, unlike immunoassays that require significant amounts of development time and rely on the availability of high-quality antibodies^7,8^, cPRISM-SRM is relatively easy to implement in research laboratories with commercially available standard instruments^32^. Therefore, in its current state, cPRISM-SRM should be useful for precise quantification of signaling pathway proteins in small numbers of cells as well as for an initial quantitative screening of many clinically important protein markers in small but important subpopulations of tumor cells (e.g., fine-needle aspirants from minimally invasive biopsies) that cannot be readily accessible by targeted antibody-based immunoassays. However, current cPRISM-SRM is still insufficient for comprehensive analysis of single mammalian cells due to its limited sensitivity and sample throughput.

Current cPRISM-SRM cannot provide sufficient sensitivity to detect and quantify the majority of proteins in a single mammalian cell. Furthermore, quantification of endogenous peptides in small numbers of cells primarily relies on a single SRM transition because other transitions either have matrix interferences or do not have sufficient SRM signal (Supplementary Figs. 3–9 and Supplementary Data 5). The single transition quantification may lack sufficient specificity and/or have the portion of SRM signal from the matrix background at low numbers of cells (Supplementary Fig. 12), resulting in the reduced quantitation accuracy. Therefore, significantly enhancing the detection sensitivity of cPRISM-SRM is necessary for moving toward targeted MS-based single-cell proteomics. The LC-MS sensitivity is known to be increased linearly with decreasing the LC flow rate, and inversely proportional to the square of the capillary LC column inner diameter (i.d.)^46,47^. When compared to state-of-the-art ultrasensitive LC-MS platforms (1–10 zmol sensitivity with an ultralow flow rate of 10–20 nL min^−1^)^11,12^, current cPRISM-SRM uses the standard nanoLC flow rate of ~400 nL min^−1^ with ~300 zmol sensitivity. Therefore, enhancing the detection sensitivity of cPRISM-SRM could be achieved by implementation of ultralow-flow capillary electrophoresis^11,14^ or LC^12,13^ without sacrificing separation efficiency when combined with advanced high-efficiency ion source and ion transmission interface^48,49^. A ~20-fold improvement in cPRISM-SRM sensitivity is expected, which could allow to detect and quantify proteins at ~10,000 copies per cell in a single mammalian cell. Based on the recent comprehensive HeLa proteome, such levels of improvement could enable quantification of ~50% human proteome in a single mammalian cell (i.e., ~7400 protein out of ~15,000 proteins in a single HeLa cell)^18^.

The other shortcoming of current cPRISM-SRM is the limited sample throughput due to the need to analyze multiple fractions for many proteins with LC-SRM individually when compared to high-throughput antibody-based immunoassays. Improving sample throughput can be achieved either by using a combination of isobaric tags and heavy isotope-labeled internal standards for sample multiplexing^50^ followed by parallel reaction monitoring on high-resolution MS or by implementing cutting-edge MS interface technologies (e.g., long-path length structures for lossless ion manipulation termed SLIM^51,52^) for ultrafast high-resolution gas-phase separation (milliseconds) without the need of the second dimensional slow LC separation (minutes). Most importantly, high-resolution PRISM fractionation is highly compatible with both strategies because it can greatly reduce the ratio compression for isobaric labeling-based targeted quantification, and PRISM and SLIM are fully orthogonal with the ability to achieve ultrahigh-resolution full-baseline separation. In contrast to our current cPRISM-SRM, ~10-fold and ~1000-fold improvement in sample throughput may be achievable for isobaric labeling and SLIM strategies, respectively.

In summary, we developed and demonstrated a facile targeted MS-based method for enabling proteomic analysis of very low numbers of mammalian cells. It can reliably detect target proteins at ≥3000 and ≥200,000 copies per cell in the equivalent of 100 cells and a single cell, respectively. Further improvement in sensitivity and throughput is needed for enabling rapid broad targeted quantification of single mammalian cells. The method can be easily implemented in MS and proteomics laboratories at no additional cost for instruments or reagents. In its current state, it should be useful for researchers to perform quantitative studies of signaling pathway proteins or a set of known proteins with important biological function in small numbers of cells or mass-limited precious clinical specimens, especially when high-quality antibodies are not available or difficult to generate for protein mutations or modifications.

## Methods

### Cell culture

HMEC line 184A1 was obtained from M. Stampfer (Lawrence Berkeley National Laboratory) and maintained in DFCI-1 medium. This medium consists of α-MEM/Ham’s nutrient mixture F-12 (1:1, vol/vol) supplemented with 12.5 ng mL^−1^ of epidermal growth factor, 10 nM triiodothyronine, 10 mM HEPES, 50 µM freshly made ascorbic acid, 2 nM estradiol, 1 µg mL^−1^ of insulin, 2.8 µM hydrocortisone, 0.1 mM ethanolamine, 0.1 mM phosphoethanolamine, 10 µg mL^−1^ of transferrin, 2 mM L-glutamine, 100 units of penicillin per ml, and 100 µg mL^−1^ of streptomycin sulfate; 15 nM sodium selenite; 1 ng mL^−1^ of cholera toxin; 1% fetal bovine serum; and 35 µg mL^−1^ of bovine pituitary extract. The pH was 7.4 at 6.5% CO_2_. Cells were plated in 15-cm dishes or 96-well plates, grown for 24 h, starved in serum-free medium for 18 h before treatment. Cells were rinsed twice with ice cold PBS and harvested in 1 mL ice cold PBS containing 1% phosphatase inhibitor cocktail (Pierce, Rockford, IL) and 10 mM NaF (Sigma Aldrich, St. Louis, MO). Cells were centrifuged at 1500 rpm for 10 min at 4 °C and excess PBS was carefully aspirated from cell pellet. Cell pellets were placed at −80 °C until further processing.

### BSA protein and its purity characterization

High-purity BSA protein at ≥99% with purification through a combination of heat shock and ethanol fractionation was purchased from Sigma-Aldrich (St. Louis, MO). Following trypsin digestion and SPE C18 cleanup, the tryptic peptides at 0.1 µg µL^−1^ were subjected to LC-MS/MS analysis to characterize the BSA purity. A nanoACQUITY UPLC system (Waters Corporation, Milford, MA) with a homemade 75 μm i.d. × 70 cm reversed-phase capillary column using 3 μm C18 particles (Phenomenex, Torrance, CA) was used for separation at a constant flow of 300 nL min^−1^ over 100 min with a gradient of 100% mobile phase A (0.1% formic acid in water) to 60% mobile phase B (0.1% formic acid in 90% acetonitrile). MS analysis was performed on a Thermo Scientific LTQ-Orbitrap Velos mass spectrometer. The heated capillary temperature and spray voltage were 350 °C and 2.2 kV, respectively. Full MS spectra were recorded at a resolution of 100,000 (*m*/*z* 400) over the range of *m*/*z* 300–2000 with an automated gain control value of 1 × 10^6^. MS/MS was performed in the data-dependent mode with an automated gain control target value of 3 × 10^4^. The most abundant 10 parent ions were selected for MS/MS using high-energy collision dissociation with a normalized collision energy setting of 30. Precursor ion activation was performed with an isolation width of 2 Da, a minimal intensity of 1000 counts, and an activation time of 100 ms.

LC-MS/MS raw data were converted into dta files using Bioworks Cluster 3.2 (Thermo Fisher Scientific, Cambridge, MA), and MSGF plus algorithm (v9979, released in March 2014) was used to search MS/MS spectra against the bovine and human protein sequence database (UniProt, released in September 2016). The key search parameters used were 20 ppm tolerance for precursor ion masses, 0.5 Da tolerance for fragment ions, partial tryptic search with up to two missed cleavages, dynamic oxidation of methionine (15.9949 Da), and static alkylation modification of Cys (57.0215 Da). Peptides were identified from database searching results applying the following criteria: *Q*-value < 0.01 and PepQ-value < 0.01. The online Thermo peptide analyzing tool was used to analyze peptide hydrophobicity.

All identified peptides and unique peptides are listed in Supplementary Data 1 and 2, respectively. Only tryptic BSA peptides (~270 unique peptides) were identified, which confirmed the high purity of BSA protein. The hydrophobicity analysis of the unique peptides has been shown in Supplementary Data 3. The BSA peptides with different hydrophobicity have broad distribution across the LC elution profile (Supplementary Fig. 1).

### Sample preparation

Cell pellets were resuspended in ice-cold cell lysis buffer (50 mM HEPES, 150 mM NaCl, 5 mM EDTA, 1% Triton X-100, pH 7.7) at a ratio of ~3:1 lysis buffer to cell pellet. Cell lysates were centrifuged at 14,000 rpm at 4 °C for 10 min and soluble protein fraction was retained. Protein concentrations were determined by the BCA assay (Pierce, Rockford, IL). Concentrated proteins, ranging from 200 to 300 µg, were denatured and reduced with 8 M urea and 10 mM DTT in 50 mM NH_4_HCO_3_ at pH 8.0 for 1 h at 37 °C. Protein cysteine residues were alkylated with 40 mM iodoacetamide for 1 h at room temperature in the dark. The resulting sample was diluted by sixfold with 50 mM NH_4_HCO_3_, pH 8.0, and digested by sequencing-grade modified porcine trypsin (Promega, Madison, WI) with a 1:50 trypsin:protein ratio (w/w) at 37 °C for overnight. The resulting digest was then desalted by using a 1 mL SPE C18 column (Supelco, Bellefonte, PA) as described previously. The final peptide concentration was determined by the BCA assay.

### HMEC cell equivalents

Typically, one mammalian cell can generate ~100 pg of the total peptides (Supplementary Table 3)^37^. A stock of 1 µg µL^−1^ of bulk HMEC cell digest was spiked into ~25 µg of BSA digest at peptide amounts of 0, 0.1, 0.5, 1, 2, 5, 10, 20, 50, and 100 ng, equivalent to 0, 1, 5, 10, 20, 50, 100, 200, 500, and 1000 HMEC cells, respectively (three replicates for 10 and 20 HMEC equivalents). Crude heavy peptide standards of EGFR pathway proteins were added to each sample with the final concentration of 2 fmol µL^−1^. The final volume of each sample was 50 µL with BSA peptide concentration at ~0.5 µg µL^−1^. To evaluate peptide recovery throughout the cPRISM-SRM workflow, another set of HMEC cell equivalents were prepared by spiking the stock of 1 µg µL^−1^ of bulk HMEC cell digest into BSA digest at concentrations of 0, 0.1, 0.5, 1, 2, 5, 10, 20, 50, and 100 ng µL^−1^ with the BSA peptide concentration at ~0.5 µg µL^−1^ used to prevent potential adsorption of cell digests to the contact surface. Crude heavy peptide standards were added to each sample with the final concentration of 100 fmol µL^−1^.

### Intact HMEC cells

A stock of ~10^4^ cells per mL (=~10^2^ cells per 10 µL) was generated with a 1:100 dilution of 10 µL of ~1 million cells per mL (=~10^4^ cells per 10 µL). Furthermore, 1 and 10 µL of the stock HMEC cells (i.e., ~10 and 100 intact HMEC cells) were added to low-binding Eppendorf tubes preloaded with ~50 µg of carrier BSA, respectively, with the final volume of 50 µL for each sample. Thus, for each intact HMEC cells the same amount of carrier BSA was used to prevent sample loss. Standard urea digestion protocol was used for sample processing without further optimization^36,37^. In all, ~50% of peptide recovery was achieved for each sample (i.e., ~25 µg of total peptides). To evaluate the entire cPRISM-SRM platform reproducibility, four replicates with 100 HMEC cells in each replicate were generated from serial dilution from HMEC cell suspension. Crude heavy peptide standards were added to each sample (the total volume of 50 µL) with the final heavy standard at 2 fmol µL^−1^ and BSA peptide concentration at ~0.5 µg µL^−1^.

### SRM assay configuration

SRM-based targeted quantification requires prior knowledge (e.g., selection of surrogate peptides and peptide transition optimization). To detect EGFR pathway proteins by SRM assays, ten tryptic peptides without miscleavage (except those peptides containing inhibitory motifs for trypsin) were initially chosen for representing each target protein based on existing LC-MS/MS results from our own laboratory and public data repositories such as PeptideAtlas, GPM, and PRIDE. For pathway proteins without existing LC-MS/MS data, in silico digestion was performed for peptide selection. All selected peptides are unique to the given proteins with no predicted posttranslational modifications. The selected peptides were further evaluated by two prediction tools: the ESP predictor^53^ and CONSeQuence software^54^. Five peptides per protein with moderate hydrophobicity, high spectral counts, and high score from the prediction tools were selected for peptide synthesis. The synthesized crude heavy isotope-labeled peptides were further evaluated for peptide response and fragmentation pattern. For each peptide, three transitions were selected on the basis of their abundances and optimal collision energy values, which is achieved by direct infusion of the individual peptides and/or multiple LC-SRM runs with collision energy ramping. A total of 2–3 peptides with the best response were selected to configure final SRM assays for each target protein (Supplementary Data 4). The potential interference for given transitions was assessed on the basis of the relative intensity ratios between the three transitions for both light and heavy peptides using a similar approach as previously reported^32,42,55^. The best transition (the one with the most intense SRM signal and without clear evidence of coeluting interference) was used to quantify the target protein in small numbers of cells (Supplementary Data 5). To calibrate the purity of crude heavy peptides, high-purity light peptides (>95%) were purchased from Thermo Scientific (San Jose, CA). The calibrated crude heavy peptide concentrations were used to calculate protein copy number across different human cell lines (Supplementary Data 6). For most proteins, high correlation between the protein copy number and the mRNA level across different cell lines was observed^37^, suggesting that the selected target peptides can represent their corresponding proteins for quantification and the L/H SRM signal should correlate well with protein concentrations (Supplementary Data 7).

### PRISM fractionation

The high pH reversed-phase LC fractionation is the main component in the PRISM workflow (Supplementary Fig. 2)^32^. A nanoACQUITY UPLC^®^ system (Waters Corporation, Milford, MA) equipped with a reversed phase capillary LC column and an autosampler was used for fractionation. Capillary reversed phase column, 200 µm i.d. × 50 cm long, were packed in-house with 3 µm Jupiter C18 bonded particles (Phenomenex, Torrence, CA). Separations were performed at mobile phase flow rates of 3.3 µL min^−1^ on the binary pump systems using 10 mM ammonium formate (pH 10) in water as mobile phase A and 10 mM ammonium formate (pH 10) in 90% acetonitrile as mobile phase B. Furthermore, ~50 µL of sample with a peptide concentration of 0.5 µg µL^−1^ and 2 fmol µL^−1^ of heavy peptide standards was typically loaded onto the reversed phase capillary column and separated using a binary gradient of 5–15% B in 15 min, 15–25% B in 25 min, 25–45% B in 25 min, 45–90% B in 38 min. After the LC separation, the eluent from the capillary column was split into two flowing streams (1:10 split) via a Tee union. A small fraction of the eluent at a flow rate of ~300 nL min^−1^ was directed to a TSQ Quantum Ultra triple quadrupole mass spectrometer (Thermo Scientific, San Jose, CA) for on-line SRM monitoring of heavy peptide standards. The TSQ Quantum Ultra instrument operating parameters were optimized for all SRM transitions by infusion of each heavy peptide. Typically, the TSQ Quantum Ultra mass spectrometer was operated with ion spray voltages of 2400 ± 100 V, a capillary offset voltage of 35 V, a skimmer offset voltage of −5 V and a capillary inlet temperature of 220 °C. The tube lens voltages were obtained from automatic tuning and calibration without further optimization. The remainder of the eluent, at a flow rate of ~3 µL min^−1^, was automatically dispensed every minute into a 96-well plate during ~100 min LC run using an automatic fraction collector (LEAP Technology, Carrboro, NC). The specific target peptide fractions were either selected based on the same elution times of heavy internal standards being monitored by the on-line SRM (i.e., intelligent selection, termed *i*Selection) or multiplexed by fraction concatenation. Prior to peptide fraction collection, 17 µL of water was added to each well of the 96-well plate to avoid the loss of peptides and dilute the peptide fraction (~1:7 dilution) for LC-SRM analysis.

### LC-SRM analysis

Following intelligent selection, target peptide fractions of interest were subjected to LC-SRM analysis (Supplementary Fig. 2). All peptide fractions were analyzed using the nanoACQUITY UPLC^®^ system (Waters Corporation, Milford, MA) coupled on-line to a TSQ Vantage triple quadrupole mass spectrometer (Thermo Scientific, San Jose, CA). The UPLC system was equipped with a nanoACQUITY UPLC BEH 1.7 µm C18 column (100 µm i.d. × 10 cm). Solvents used were 0.1% formic acid in water (mobile phase A) and 0.1% formic acid in 90% acetonitrile (mobile phase B). Furthermore, ~16 µL of each PRISM fraction sample rather than typical ~4 µL were loaded onto the column with newly installed 20 µL of loop at a flow rate of 1 µL min^−1^ for 40 min to significantly increase detection sensitivity (Supplementary Fig. 17**)**. For the non-PRISM fractionated samples (i.e., small numbers of HMEC cell equivalents with 100 fmol µL^−1^ of heavy peptide standards), ~1 µL was loaded onto the column using 5 µL of loop at a flow rate of 0.5 µL min^−1^ for 13 min. Peptide separations were performed at a flow rate of 400 nL min^−1^ using an ACQUITY UPLC BEH 1.7 µm C18 column (100 µm i.d. × 10 cm), which was connected to a chemically etched 20 µm i.d. fused-silica emitter via a conductive carbon fiber peek union (for avoiding phosphorylated peptide loss during electrospray ionization). Peptides were separated at a flow rate of 400 nL min^−1^, using a binary gradient of 5–20% B in 26 min, 20–25% B in 10 min, 25–40% B in 8 min, 40–95% B in 1 min and at 95% B for 7 min for a total of 52 min and the analytical column was re-equilibrated at 99.5% A for 8 min. The TSQ Vantage was operated in the same manner as the TSQ Quantum Ultra. A dwell time of 30 ms was used for all SRM transitions.

### Data analysis

SRM data analysis is similar as previously described^32^. SRM data acquired on the TSQ Vantage were analyzed using Skyline software. Matrix inferences from co-eluting peptides with a transition that falls within the mass width of Q1 and Q3 were detected by deviation from the expected L/H SRM signal ratios. The best transition for each peptide was used for quantification, and the L/H SRM ratios were used to generate calibration curves. Peak detection and integration were determined based on two criteria: (1) the same retention time; (2) approximately the same relative SRM peak intensity ratios across multiple transitions between light peptides and heavy peptide standards. All data were manually inspected to ensure correct peak detection and accurate integration. The S/N ratio was calculated by peak intensity at the apex over the average background noise in a retention time window of ± 15 s for the target peptides. The background noise levels were conservatively estimated by visual inspection of the chromatographic peak regions. The LOD and LOQ were defined as the lowest concentration points of each target protein at which the S/N of surrogate peptides was at least 3 and 7, respectively. For conservatively determining the LOQ values, in addition to the requirements of the S/N to equal or be above 7, one additional criteria was applied: surrogate peptide response over the protein concentration be within the linear dynamic range. All calibration and correlation curves were plotted using Microsoft Excel 2007. The RAW data from TSQ Vantage were loaded into Skyline software to display graphs of XICs of multiple transitions of target proteins monitored. Standard derivation and CV were calculated based on three or four processing replicates of cPRISM-SRM measurements.

### Data availability

All the Skyline-processed SRM results reported in this study can be accessed at Panorama without restrictions (Access link: https://panoramaweb.org/UFRQg2.url).

## Electronic supplementary material

Supplementary file

Description of Supplementary Files

Supplementary Data 1

Supplementary Data 2

Supplementary Data 3

Supplementary Data 4

Supplementary Data 5

Supplementary Data 6

Supplementary Data 7
