# Supplementary material for: Facile carrier-assisted targeted mass spectrometric approach for proteomic analysis of low numbers of mammalian cells
Source: Commun Biol. 2018 Aug 6;1:103. doi: 10.1038/s42003-018-0107-6 (PMC6123794; doi:10.1038/s42003-018-0107-6)
Supplement: Supplementary file 1 — Supplementary file [file 42003_2018_107_MOESM1_ESM.pdf]

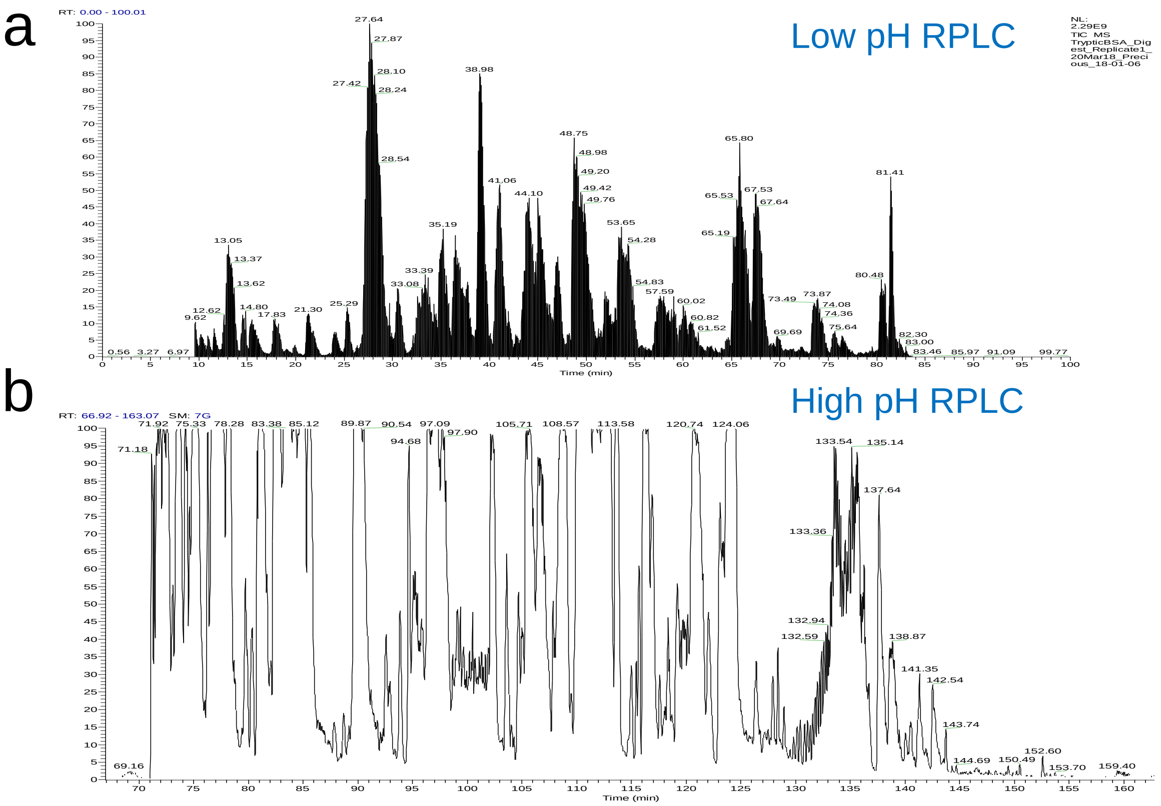

Supplementary Figure 1. (a) Global LC-MS/MS analysis of BSA tryptic digest with low pH RPLC separation coupled to LTQ-Orbitrap Velos MS with 100-min LC gradient time. **(b)** Full MS scan of BSA tryptic digest with high pH RPLC separation coupled to QQQ MS with 120-min LC gradient time. BSA tryptic peptides cover the entire LC profile in both low and high pH RPLC separations.

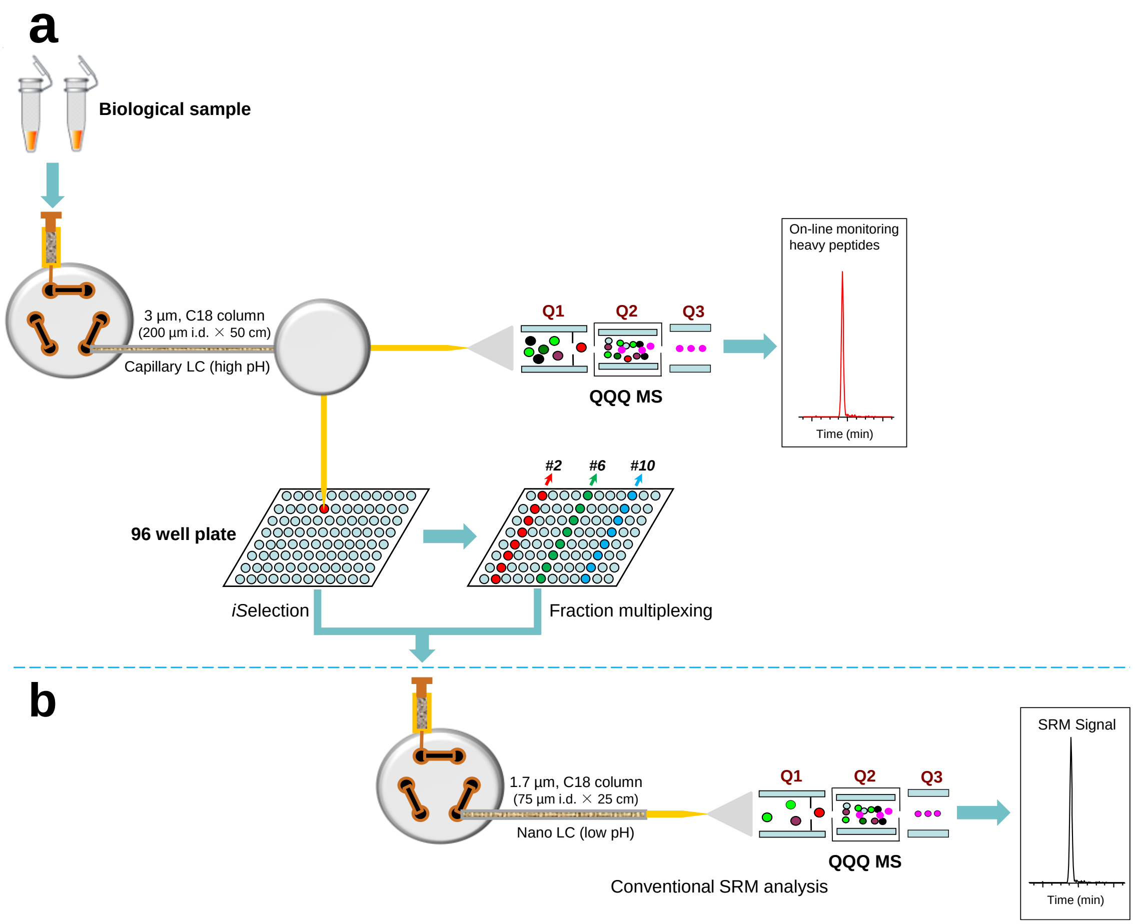

Supplementary Figure 2. Schematic diagram of the PRISM-SRM workflow (Shi *et al.* PNAS 109:15395-15400, 2012). (**a**) PRISM workflow. ~25 µg peptide sample spiked with internal standard (IS) heavy peptides was injected and separated by a high resolution reversed-phase cLC system using high pH mobile phases. The eluent from the cLC column at a flow rate of 3.3 µL/min was split into two flowing streams via a Tee union (the split ratio of flow rates is 1:10): a small fraction (9%) of the column eluent went to a triple quadrupole mass spectrometer for on-line SRM monitoring IS peptides; a large fraction (91%) of the column eluent was automatically collected every minute into a 96-well plate during a ~100-min LC run. The specific target peptide fractions were selected based on the same elution times of IS being monitored by the on-line SRM (i.e., intelligent selection, termed *i*Selection) or multiplexed. (**b**) Conventional LC-SRM workflow. Following *i*Selection, a target peptide fraction with the total volume of ~20 µL was directly subjected to nanoLC-SRM with 16 µL sample per injection prior to nanoLC-SRM analysis. Based on our recent studies PRISM-SRM can provide ~200-fold improvement in sensitivity with the quantitation dynamic range of ~7 orders of magnitude when compared to regular LC-SRM. Its processing reproducibility has an average CV of ~10% (Shi *et al.* PNAS 109:15395-15400, 2012; J Proteome Res 12:3353-3361, 2013; Anal Chem 89:9139-9146, 2017).

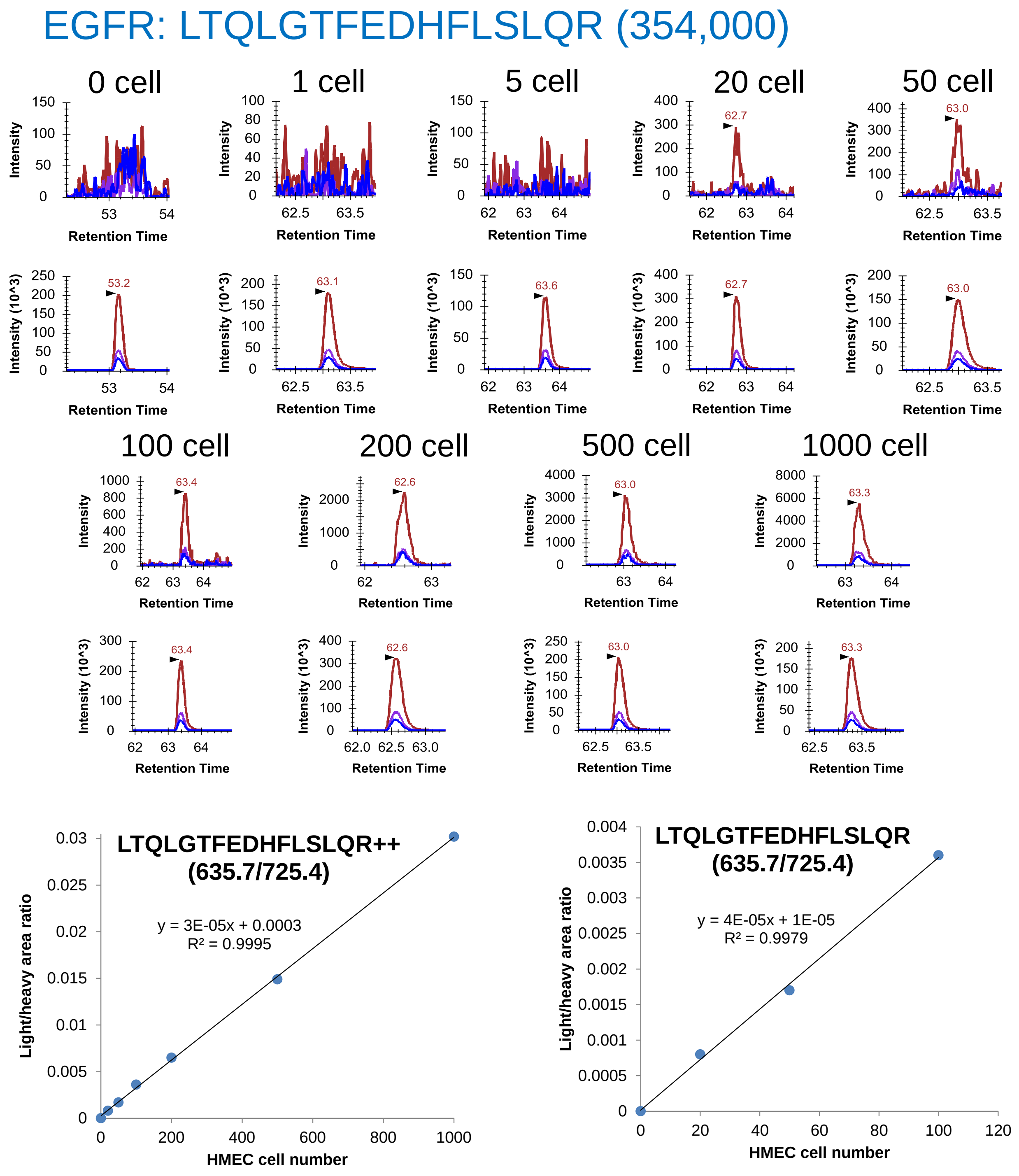

Supplementary Figure 3. Extracted ion chromatograms (XICs) and calibration curves of LTQLGTFEDHFLSLQR derived from EGFR in HMEC cell equivalents at the range of 0-1000. The best interference-free transition was used for generating calibration curves.

H/K/NRAS: LVVVGAGGVGK (213,232)

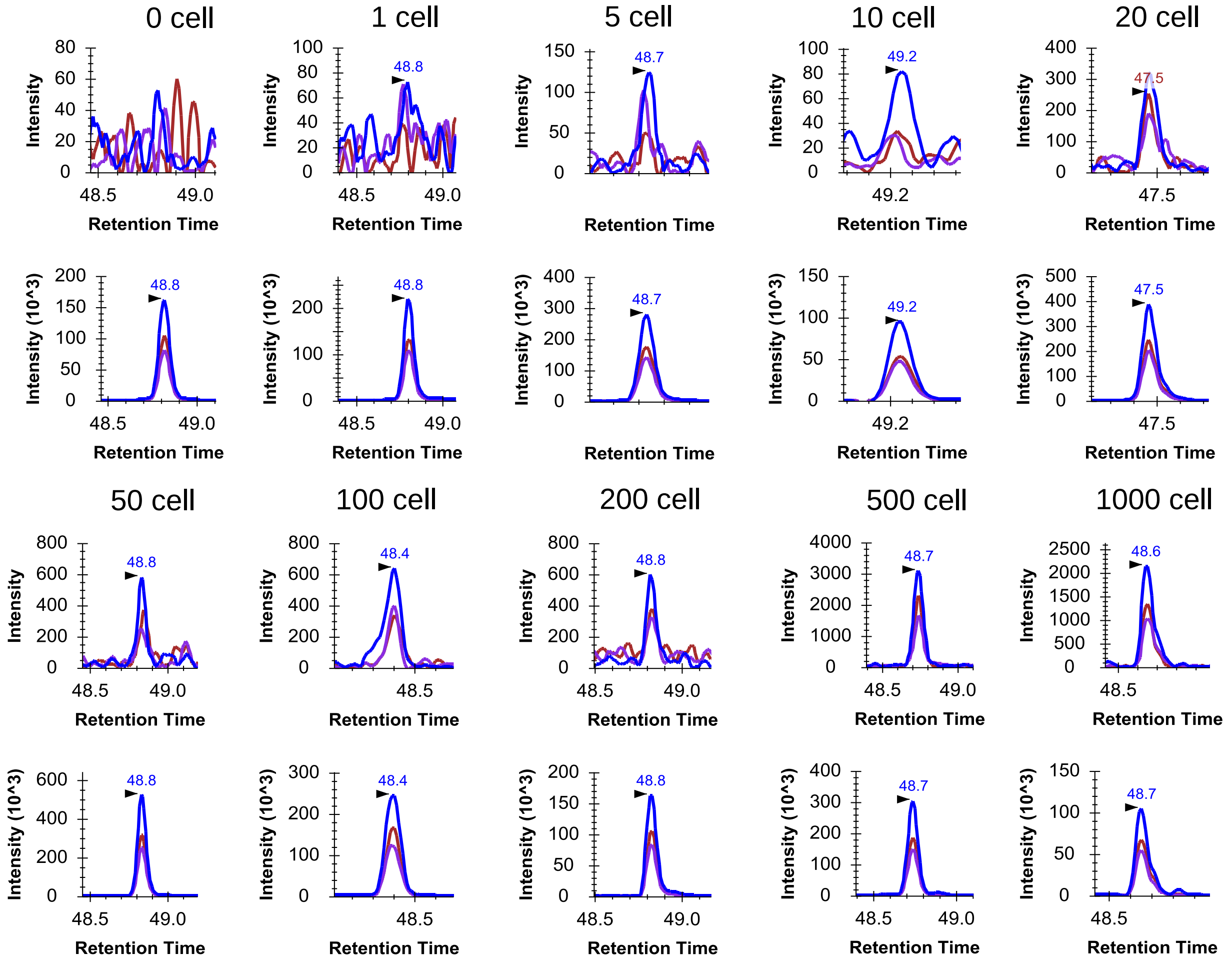

H/K/NRAS: LVVVGAGGVGK (213,232)

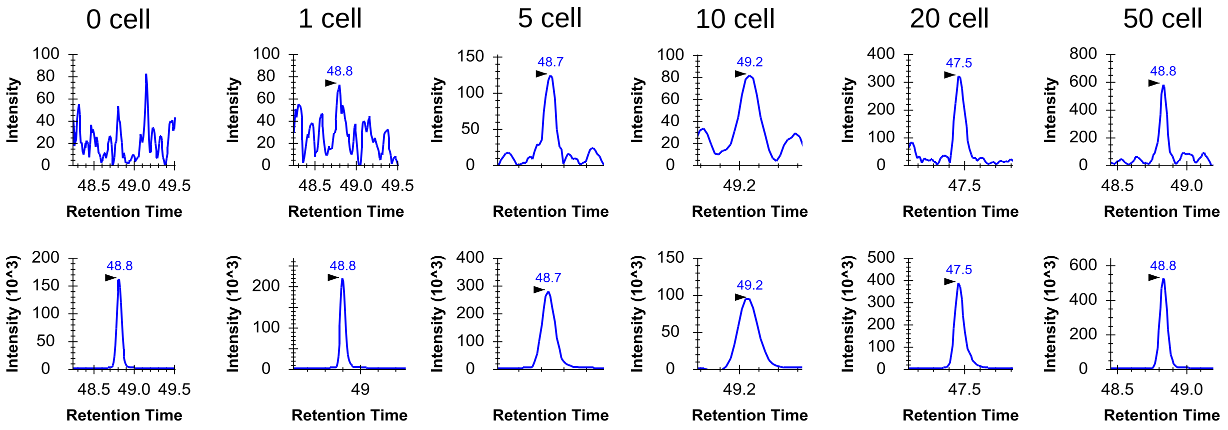

*The best interference-free transition.

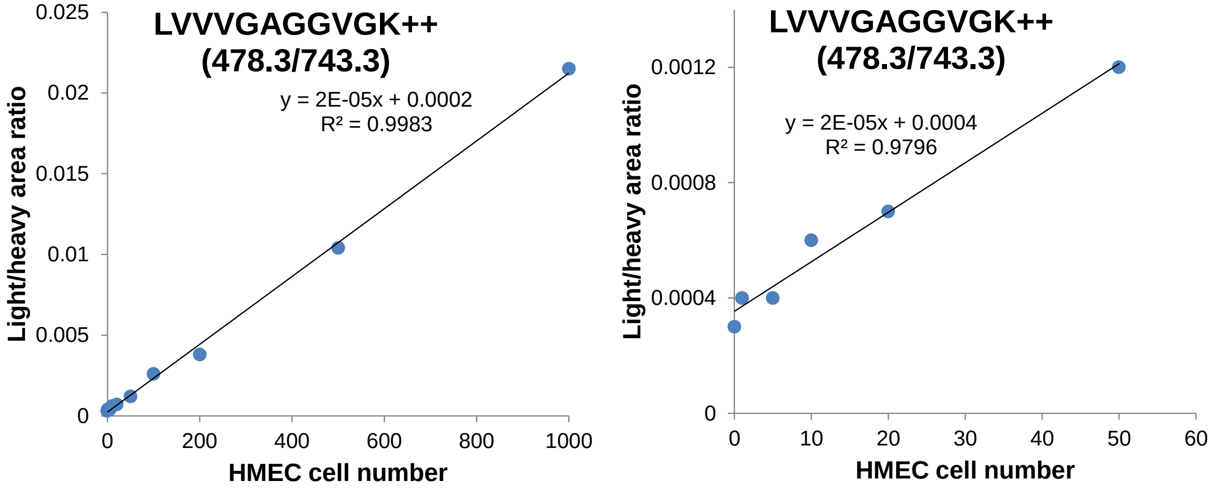

Supplementary Figure 4. XICs and calibration curves of LVVVGAGGVGK derived from H/K/NRAS in HMEC cell equivalents at the range of 0-1000. The best interference-free transition was used for generating calibration curves.

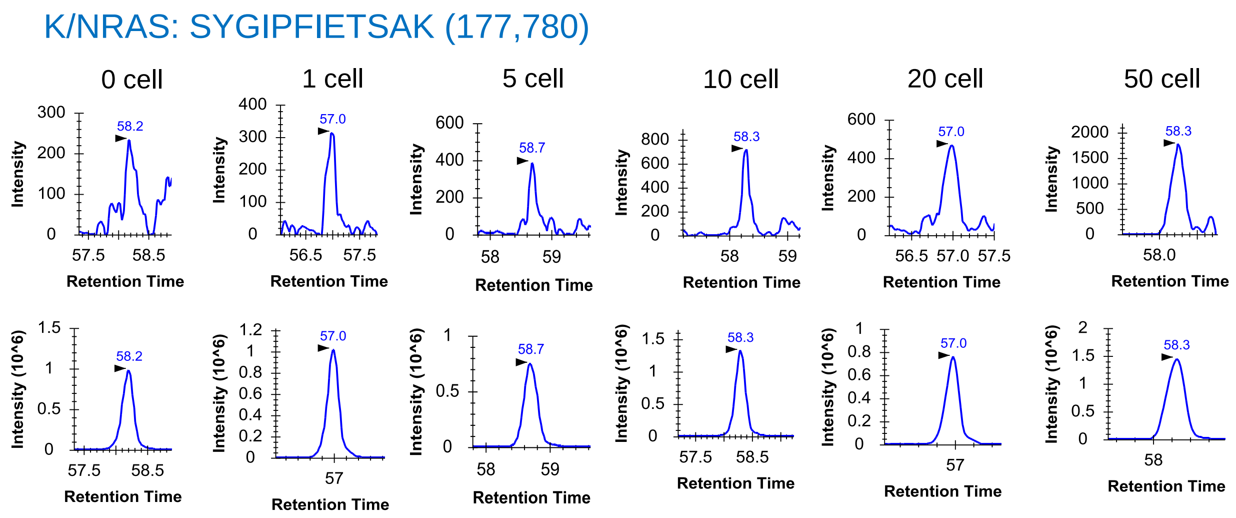

*The best interference-free transition.

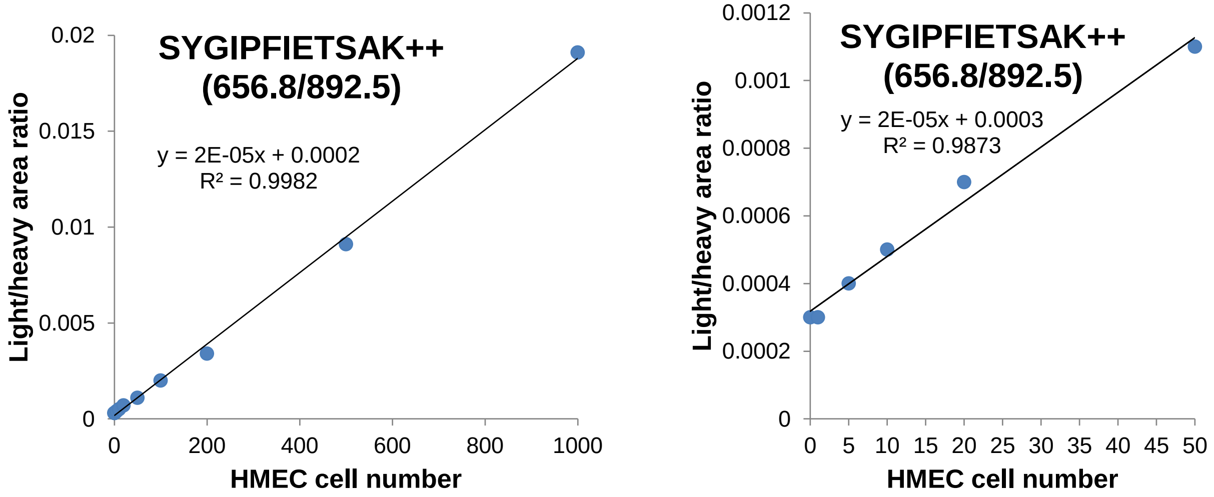

Supplementary Figure 5. XICs and calibration curves of SYGIPFIETSAK derived from K/NRAS in HMEC cell equivalents at the range of 0-1000. The best interference-free transition was used for generating calibration curves.

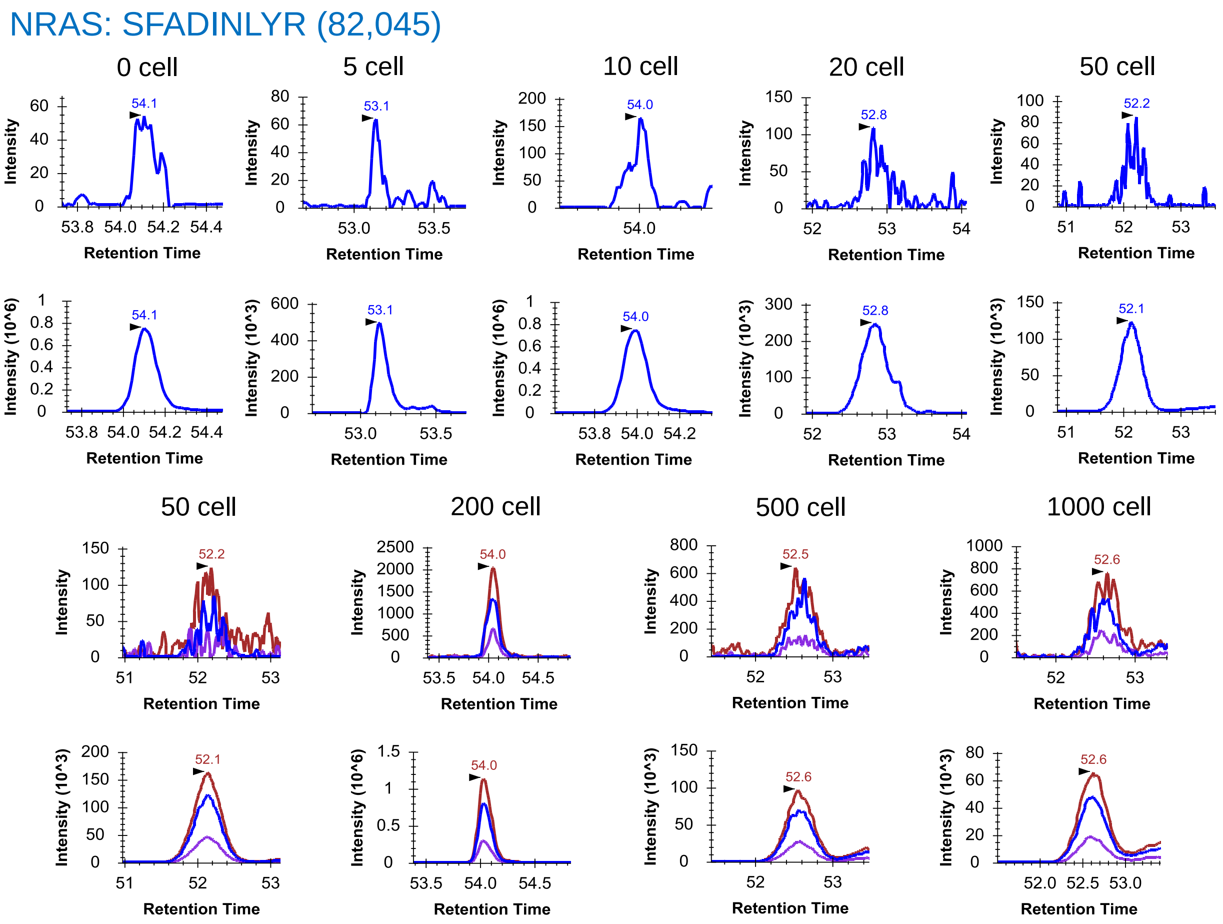

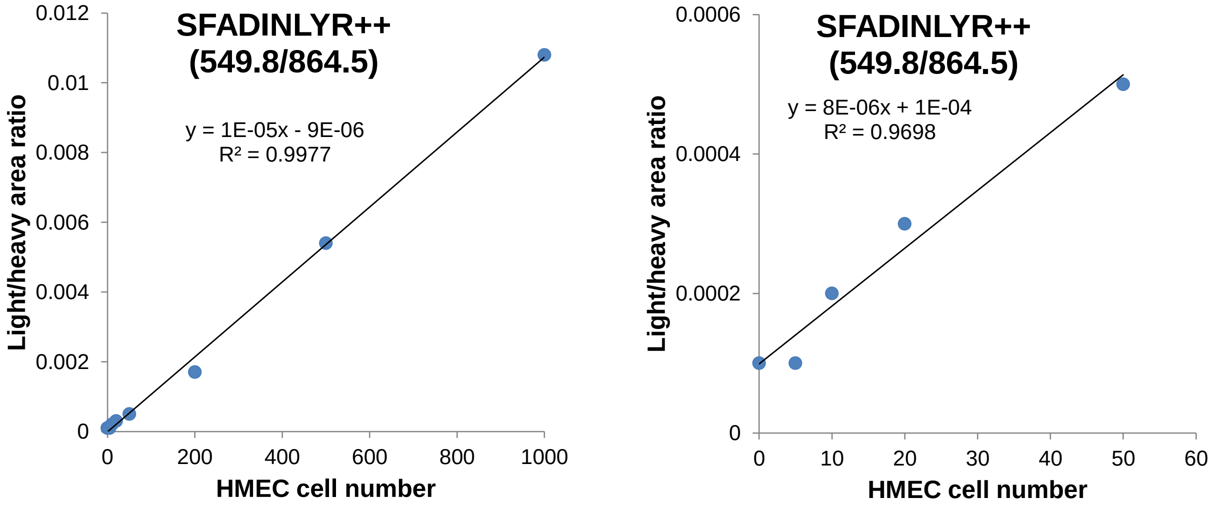

Supplementary Figure 6. XICs and calibration curves of SFADINLYR derived from NRAS in HMEC cell equivalents at the range of 0-1000. The best interference-free transition was used for generating calibration curves.

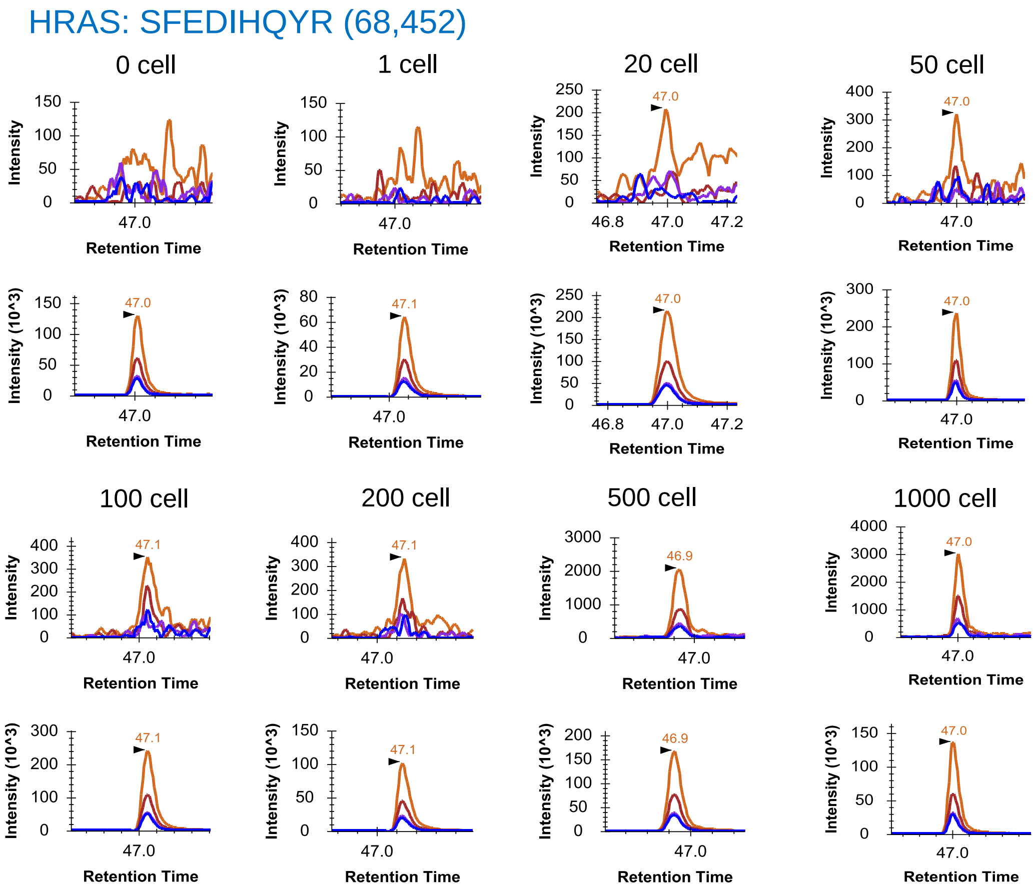

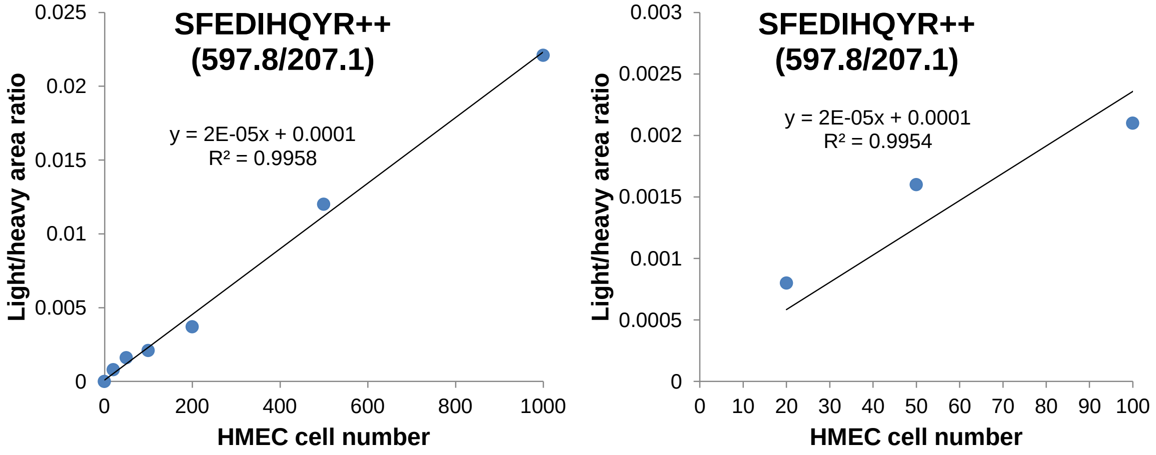

Supplementary Figure 7. XICs and calibration curves of SFEDIHQYR derived from HRAS in HMEC cell equivalents at the range of 0-1000. The best interference-free transition was used for generating calibration curves.

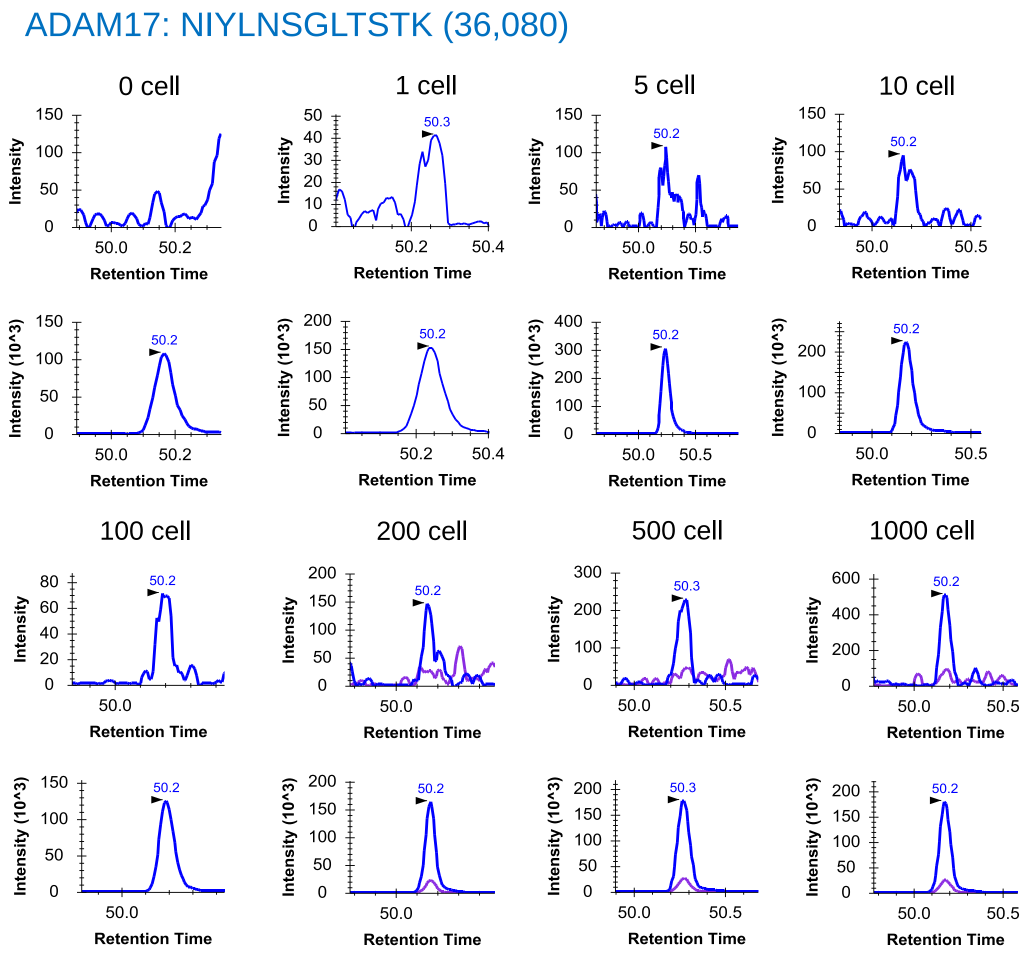

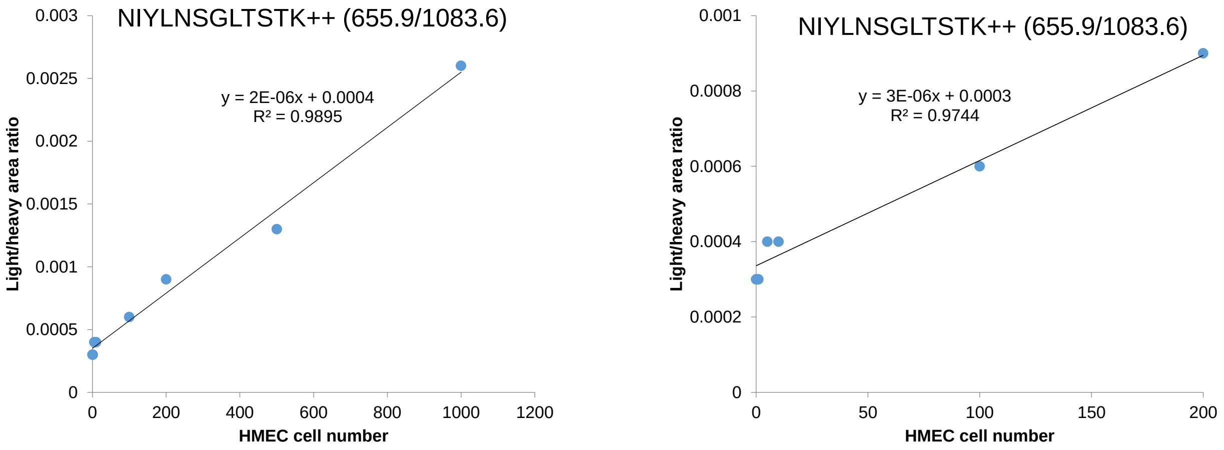

Supplementary Figure 8. XICs and calibration curves of NIYLNSGLTSTK derived from ADAM17 in HMEC cell equivalents at the range of 0-1000. The best interference-free transition was used for generating calibration curves.

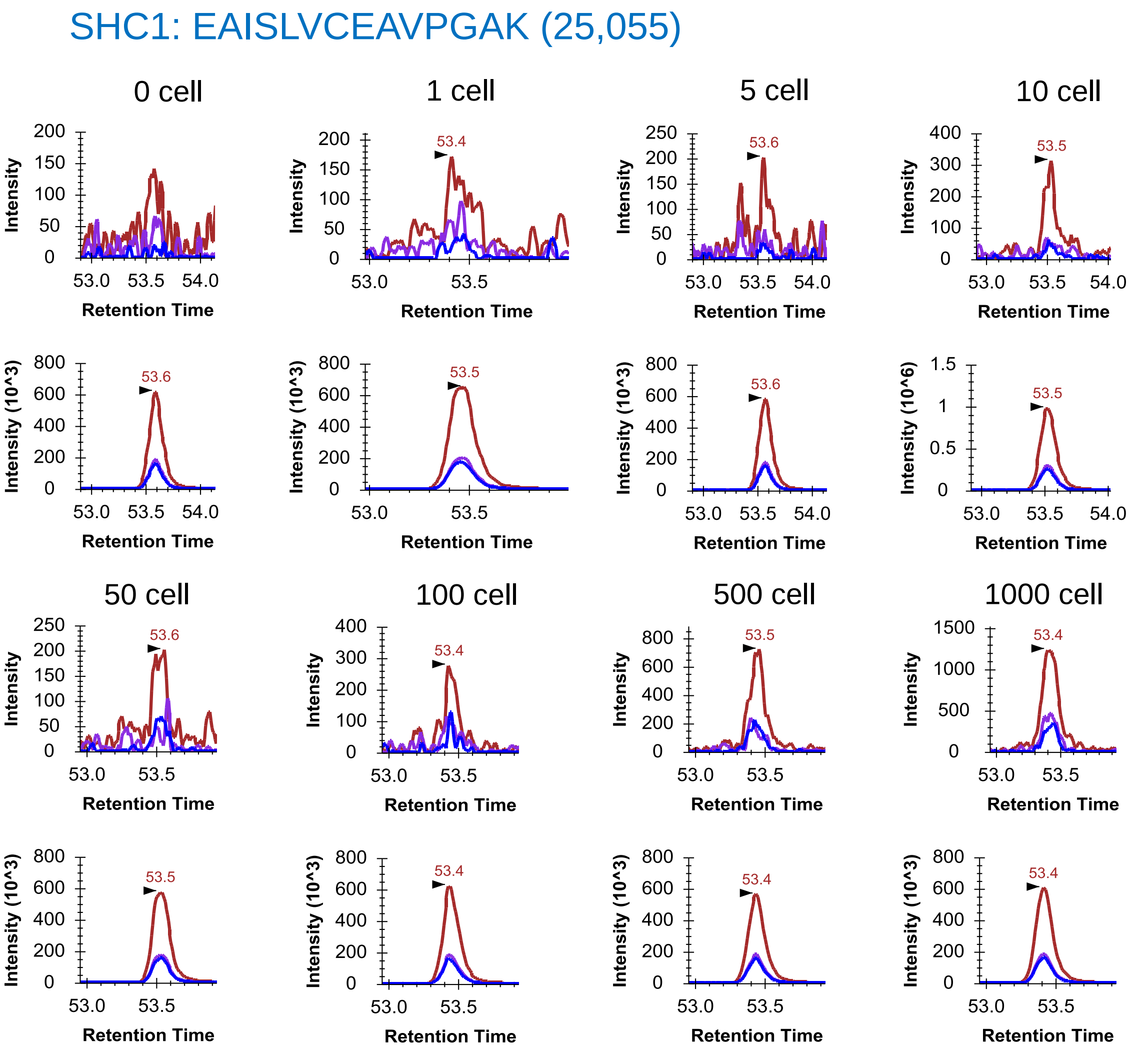

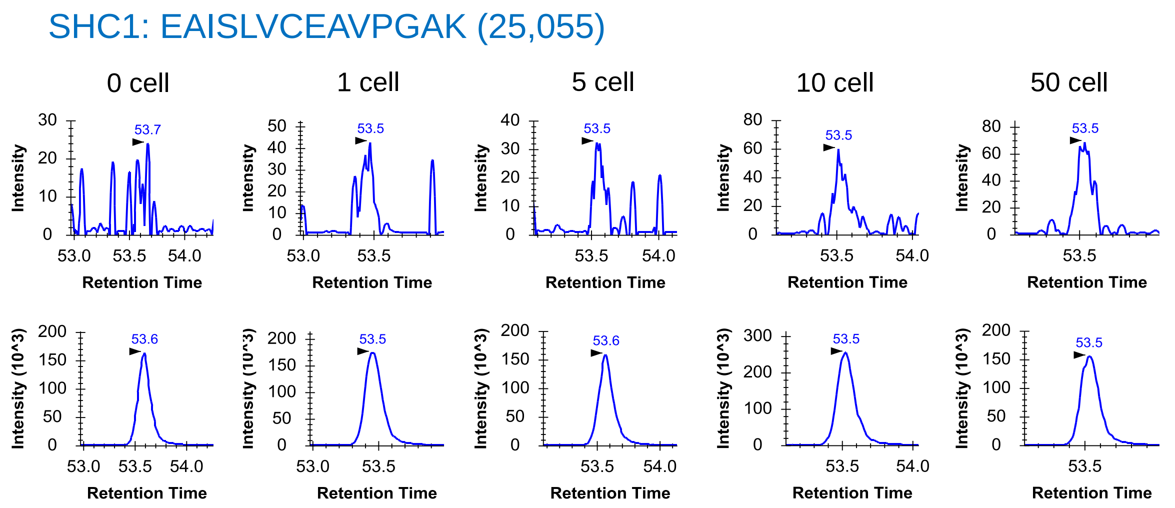

*The best interference-free transition.

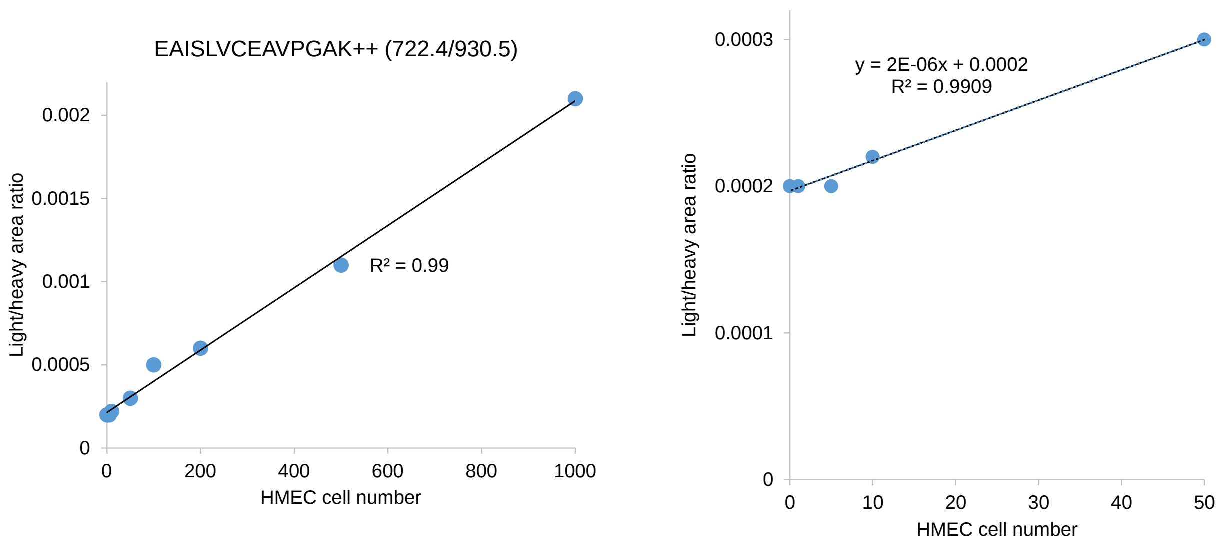

Supplementary Figure 9. XICs and calibration curves of EAISLVCEAVPGAK derived from SHC1 in HMEC cell equivalents at the range of 0-1000. The best interference-free transition was used for generating calibration curves.

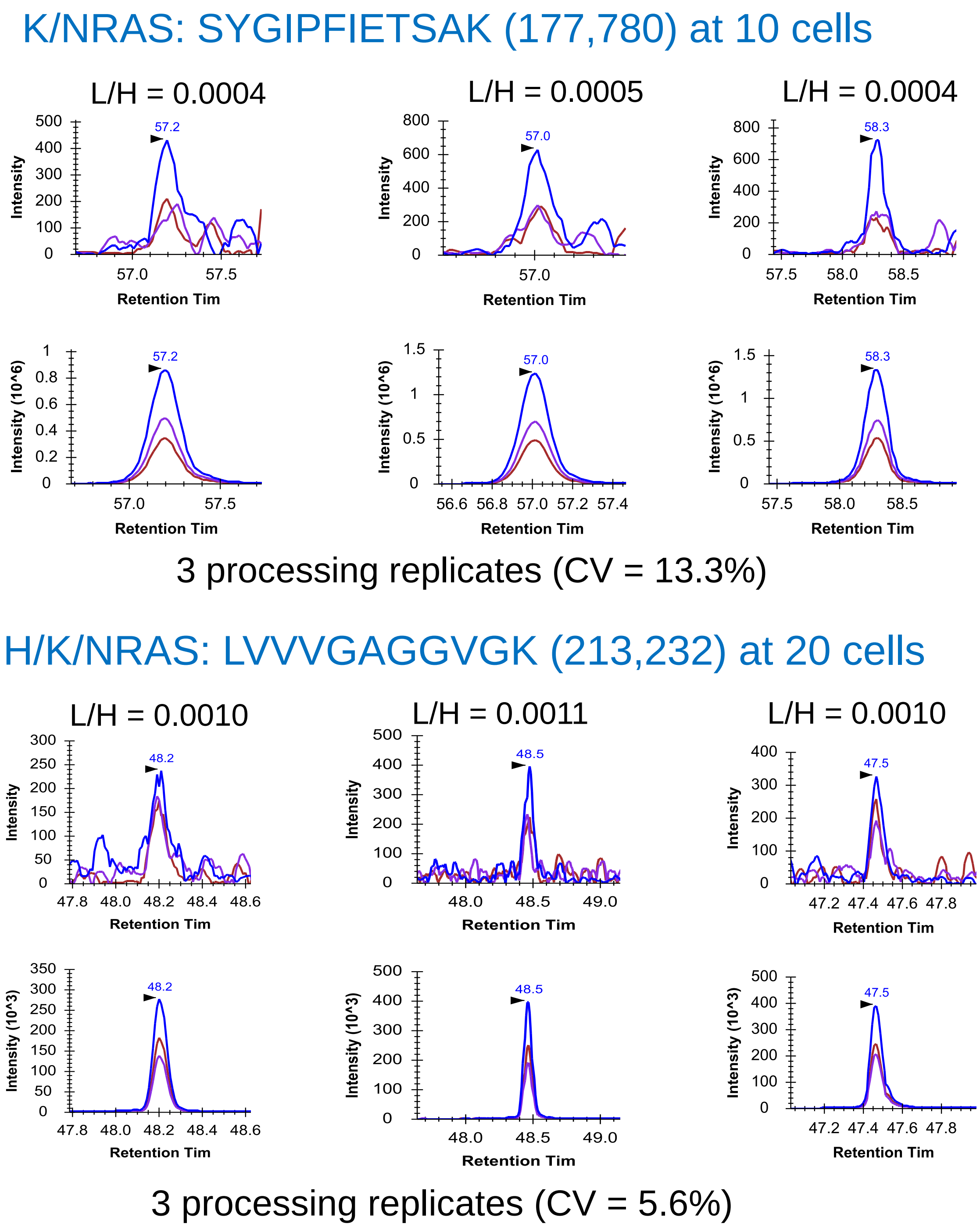

Supplementary Figure 10. Evaluation of cPRISM-SRM reproducibility with three processing replicates for SYGIPFIETSAK derived from K/NRAS at 10 cell equivalents and LVVVGAGGVGK derived from H/K/NRAS at 20 cell equivalents.

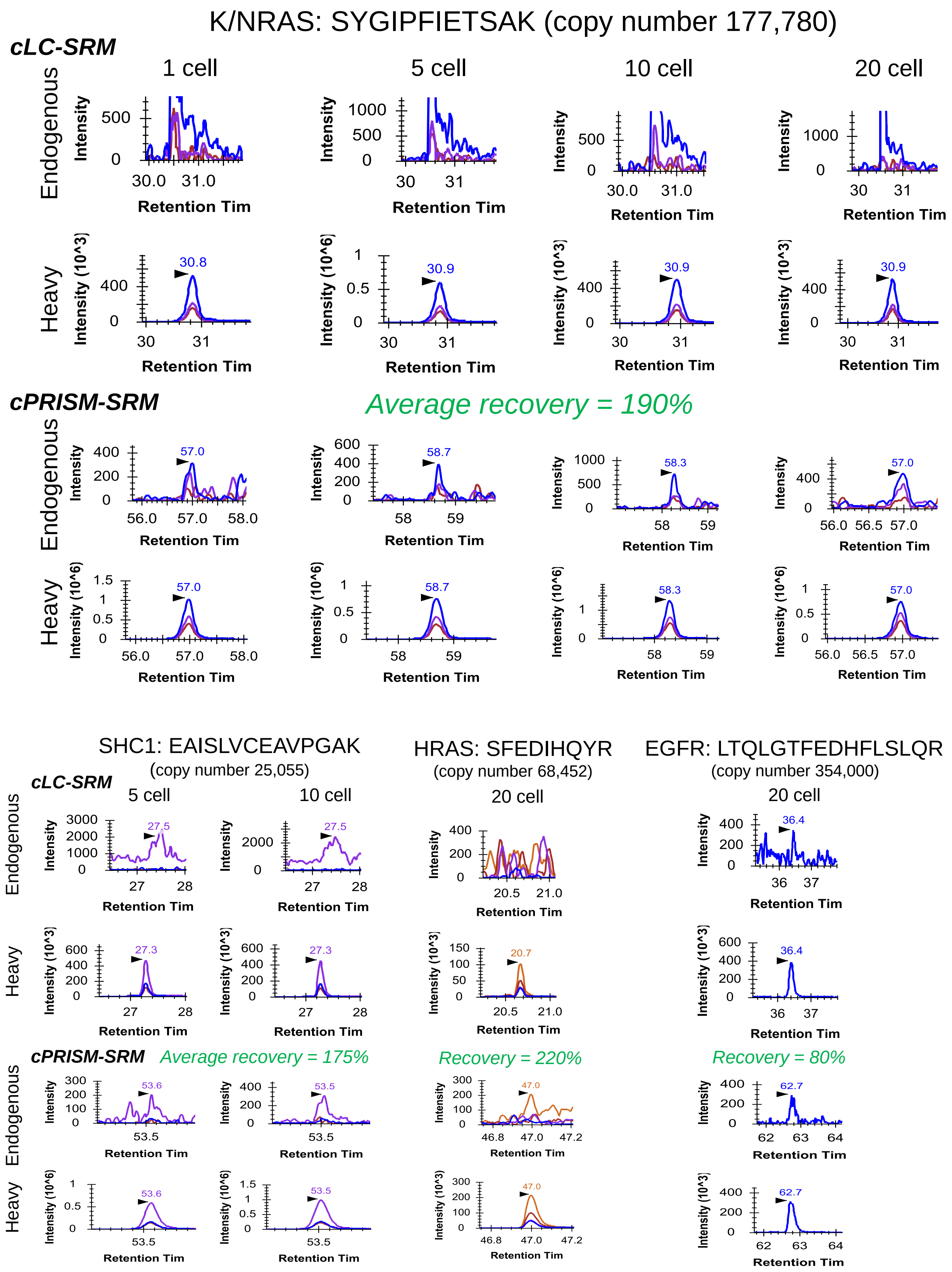
K/NRAS: SYGIPFIETSAK (copy number 177,780)
cLC-SRM
1 cell
5 cell
10 cell
20 cell
Endogenous
Heavy
Intensity
Intensity (10^3)
Intensity (10^6)
Retention Tim
cPRISM-SRM
Average recovery = 190%
SHC1: EAISLVCEAVPGAK
(copy number 25,055)
HRAS: SFEDIHQYR
(copy number 68,452)
EGFR: LTQLGTFEDHFLSLQR
(copy number 354,000)
Average recovery = 175%
Recovery = 220%
Recovery = 80%

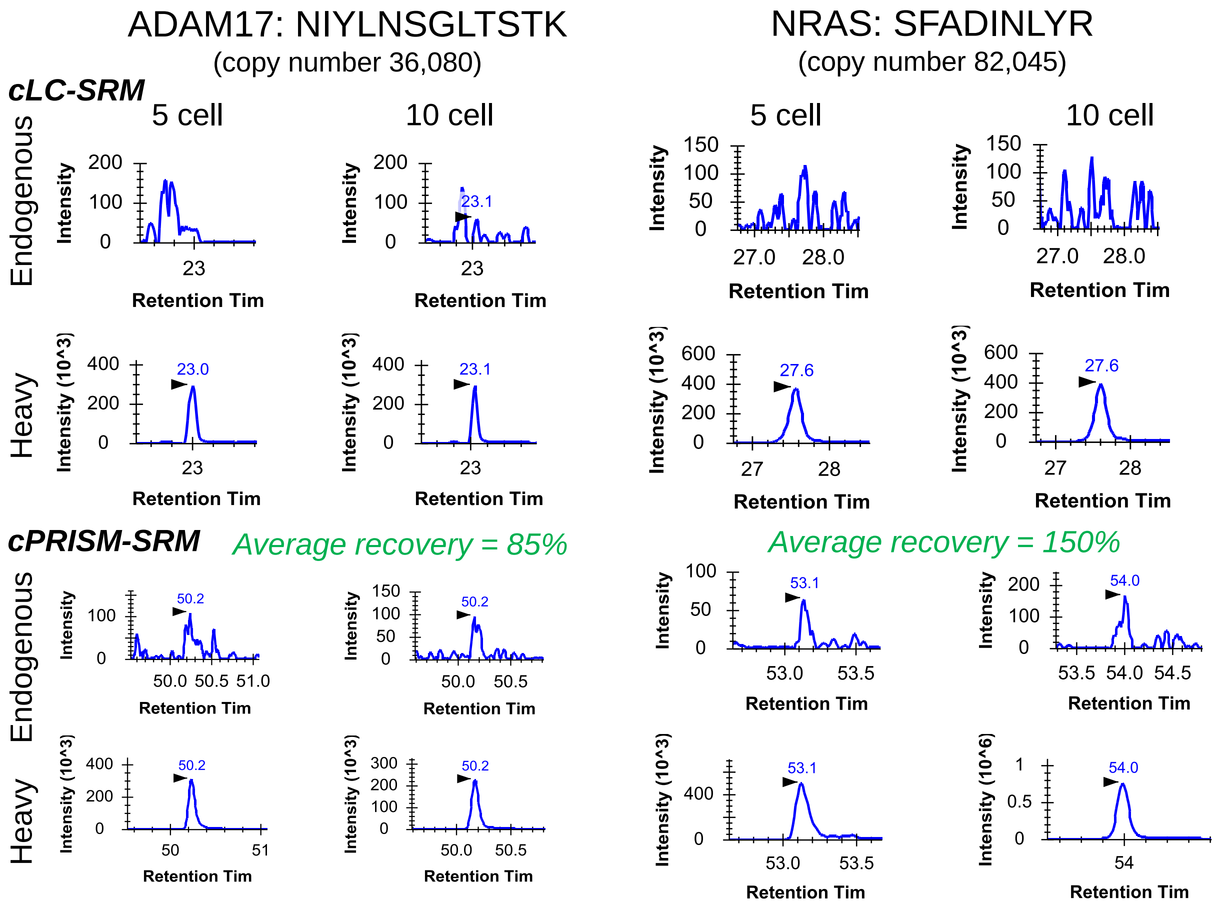

Supplementary Figure 11. Evaluation of the peptide recovery at small numbers of cells (1-20 cell equivalents) throughout the cPRISM-SRM workflow when compared to carrier-assisted LC-SRM (cLC-SRM) assuming ~100% recovery for LC-SRM with direct injection. The same amount of heavy internal standards (i.e., 100 fmol) and the same number of HMEC cell equivalents were used for both cPRISM-SRM and cLC-SRM analysis. The overall peptide recovery from cPRISM-SRM is equal to the SRM signal of heavy internal standards from cPRISM-SRM over that from LC-SRM. High recovery suggested less ion suppression in cPRISM-SRM than LC-SRM due to high-resolution PRISM separation for target peptide enrichment. Since the heavy internal standards essentially have the same physical and chemical properties as their corresponding light peptides with the only difference in mass, the endogenous light peptides in small numbers of cells are expected to have the same peptide recovery as the heavy internal standards at small numbers of cells.

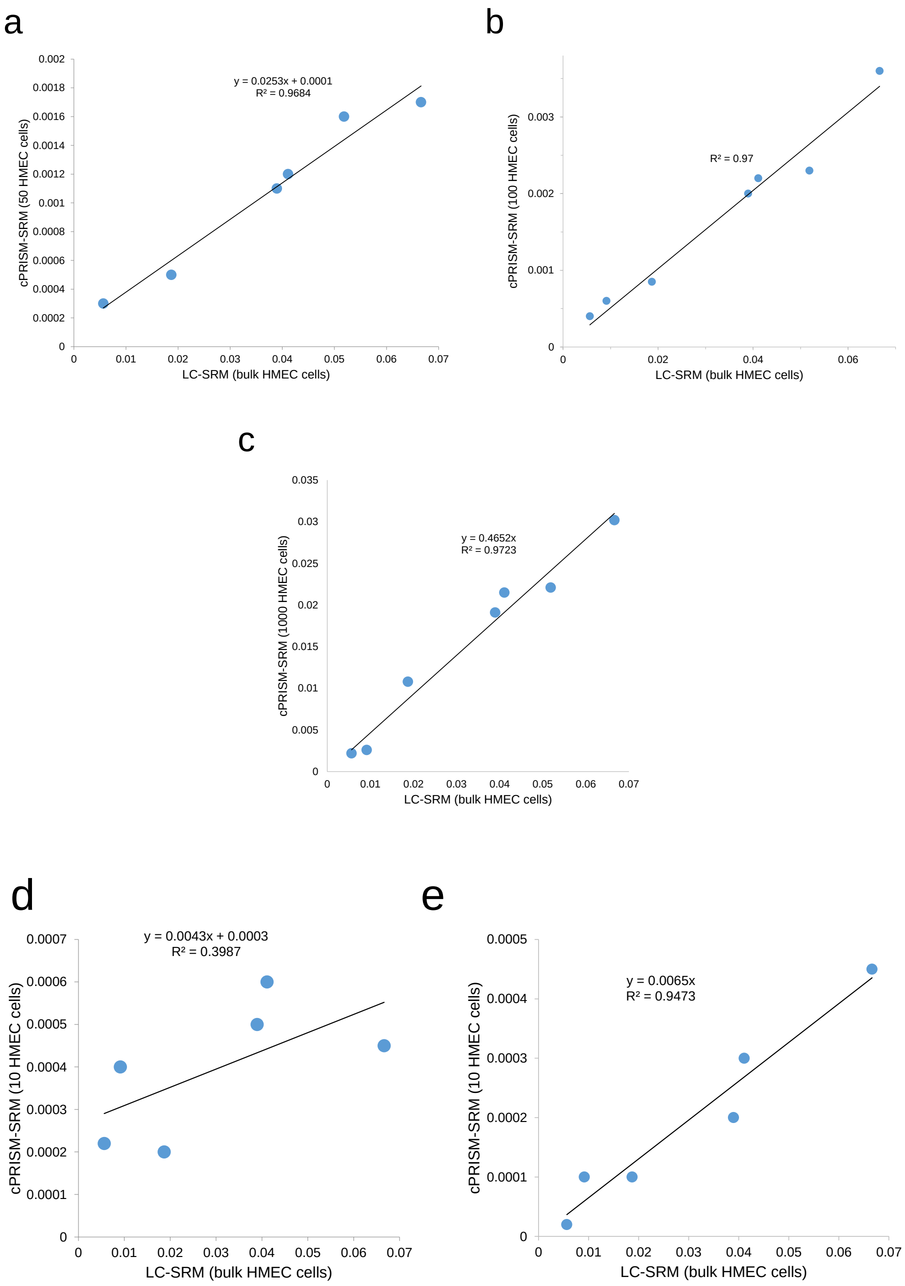

Supplementary Figure 12. Correlation analysis of targeted quantification of EGFR pathway proteins between a small number of HMEC cell equivalents with cPRISM-SRM and bulk HMEC cells with LC-SRM to evaluate measurement accuracy of cPRISM-SRM for a small number of cells. (**a**) 50 HMEC cell equivalents. (**b**) 100 HMEC cell equivalents. (**c**) 1000 HMEC cell equivalents. (**d**) 10 HMEC cell equivalents. (**e**) 10 HMEC cell equivalents with the subtraction of the background SRM signal from the blank sample.

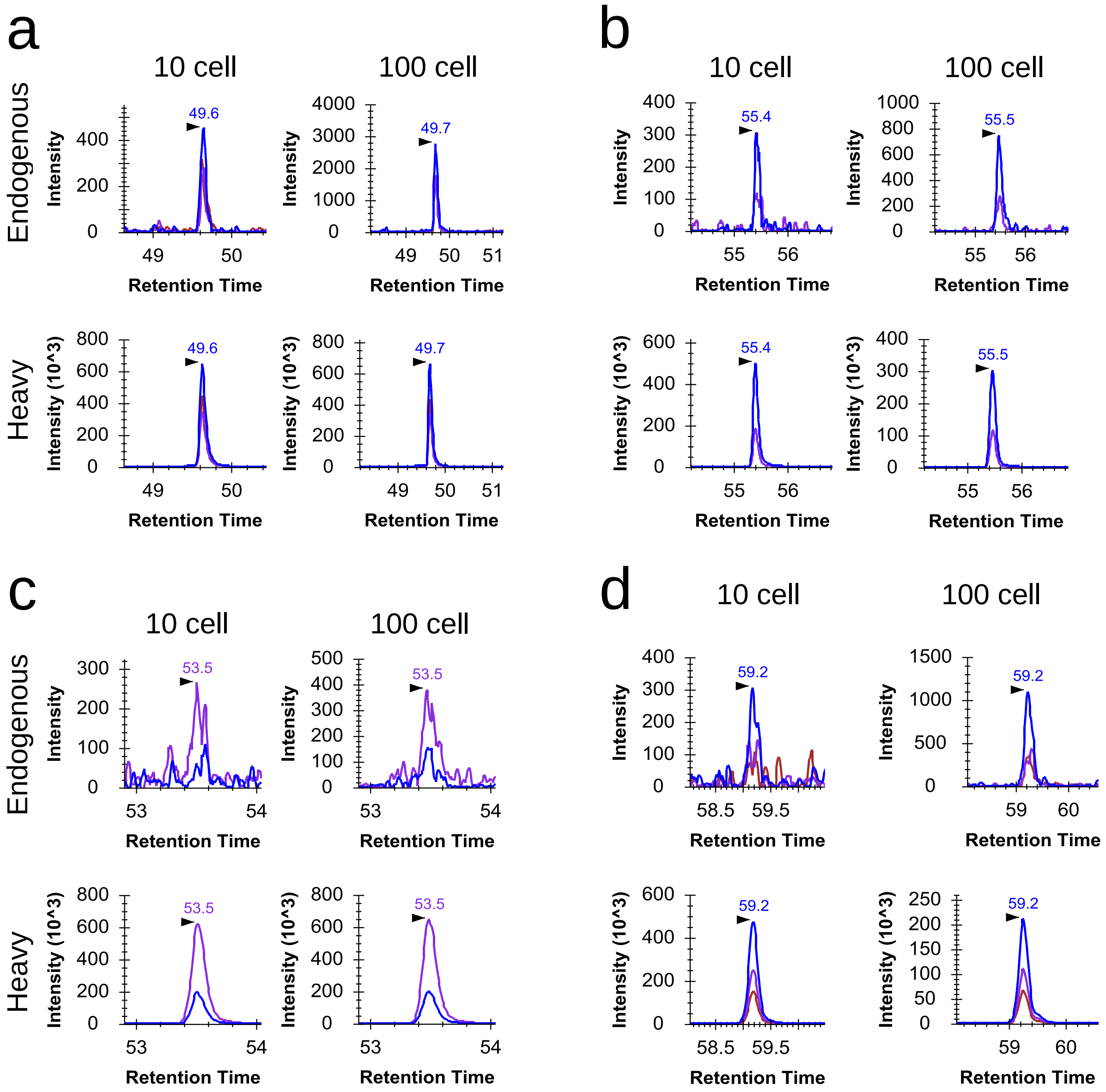

Supplementary Figure 13. Comparison of SRM signal between 10 and 100 intact HMEC cells measured by cPRISM-SRM. (**a**) XICs of transitions monitored for LVVVGAGGVGK derived from H/K/NRAS. (**b**) XICs of transitions monitored for SFADINLYR derived from NRAS. (**c**) XICs of transitions monitored for EAISLVCEAVPGAK derived from SHC1. (**d**) XICs of transitions monitored for SYGIPFIETSAK derived from K/NRAS.

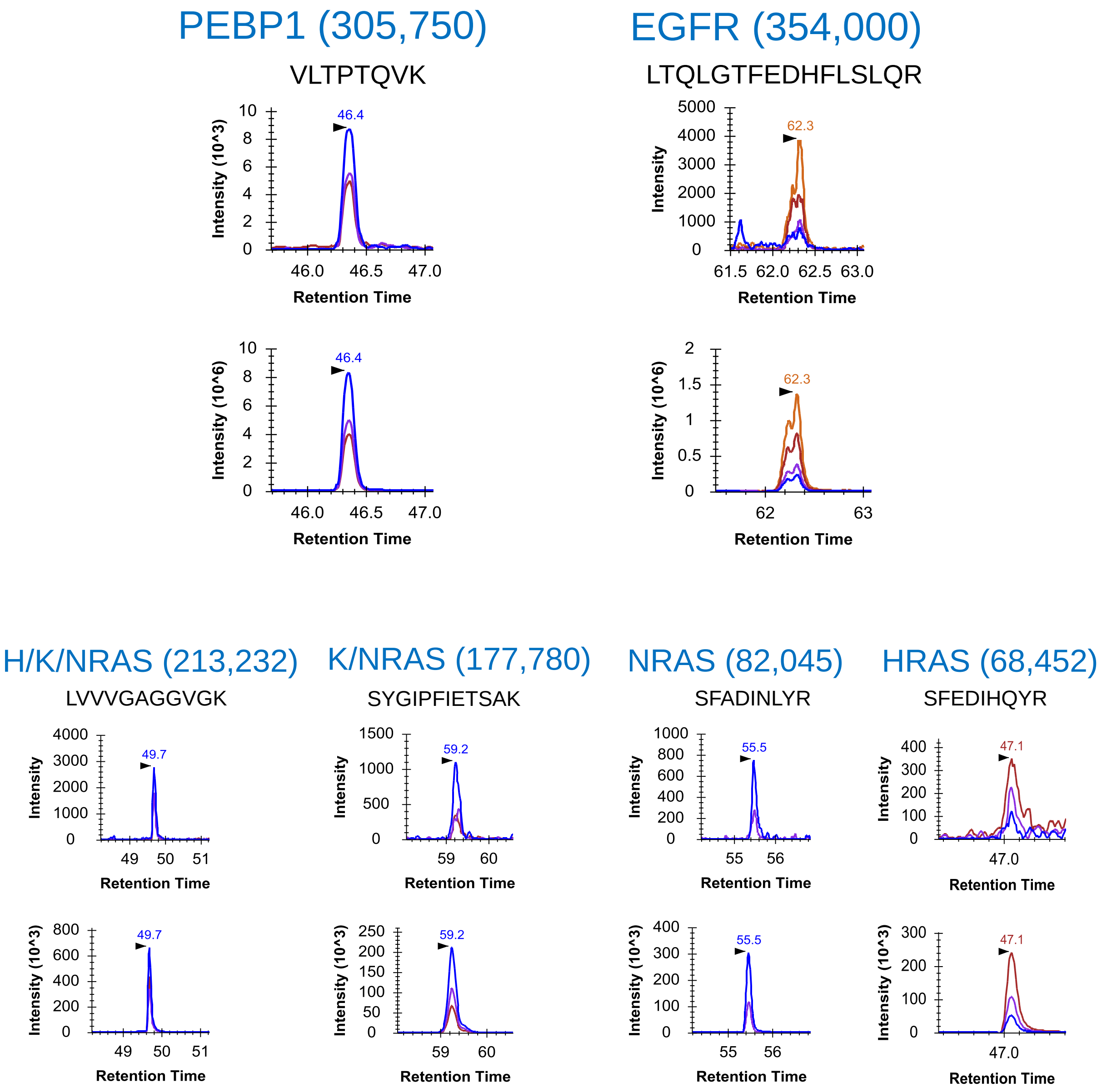
PEBP1 (305,750)
VLTPTQVK
EGFR (354,000)
LTQLGTFEDHFLSLQR
H/K/NRAS (213,232)
LVVVGAGGVGK
K/NRAS (177,780)
SYGIPFIETSAK
NRAS (82,045)
SFADINLYR
HRAS (68,452)
SFEDIHQYR
Intensity
Intensity (10^3)
Intensity (10^6)
Retention Time
46.4
62.3
49.7
59.2
55.5
47.1

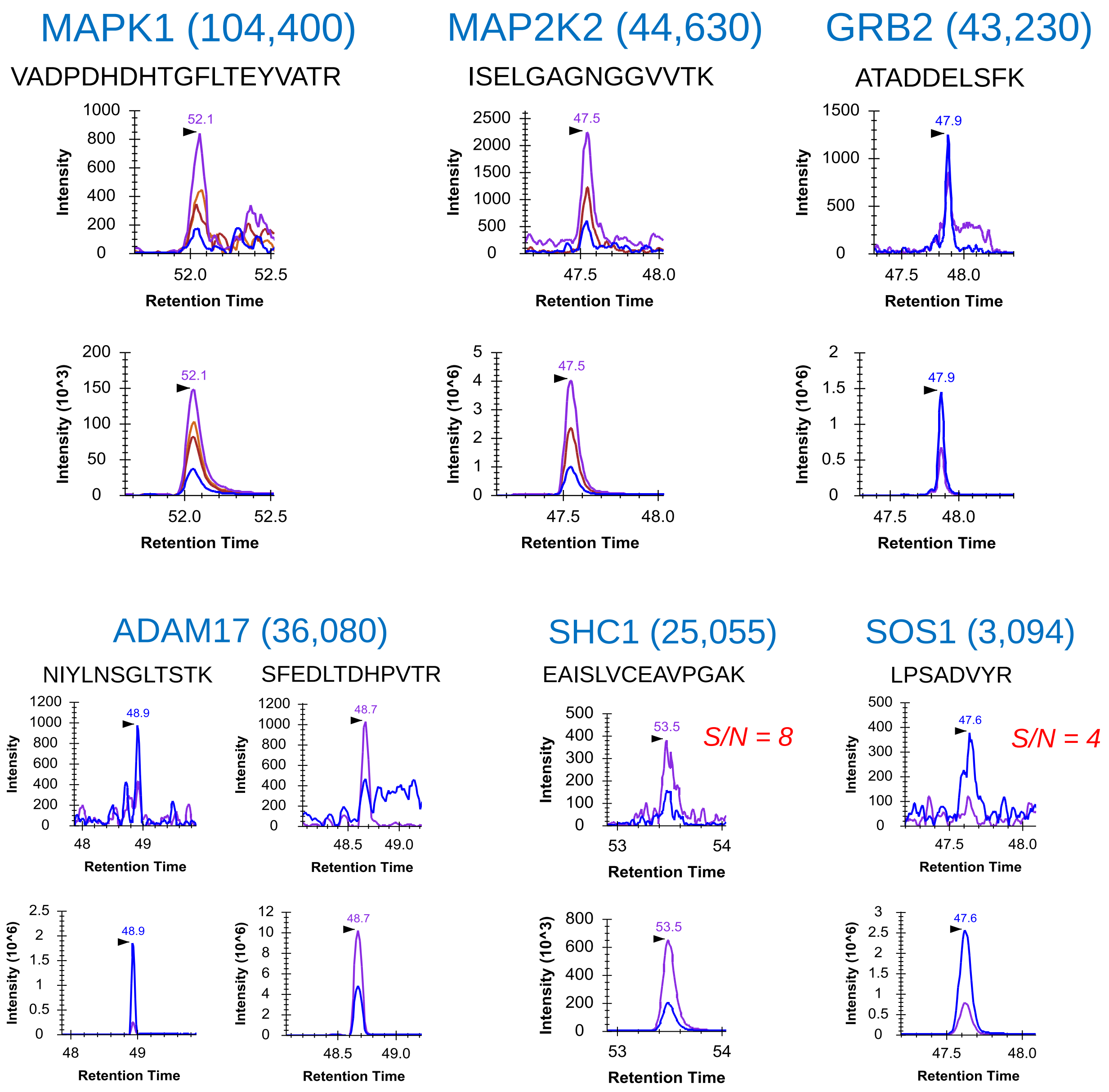

Supplementary Figure 14. XICs of detected EGFR pathway proteins in intact 100 HMEC cells by cPRISM-SRM (SOS1 protein with ~3000 copies per cell was detected).

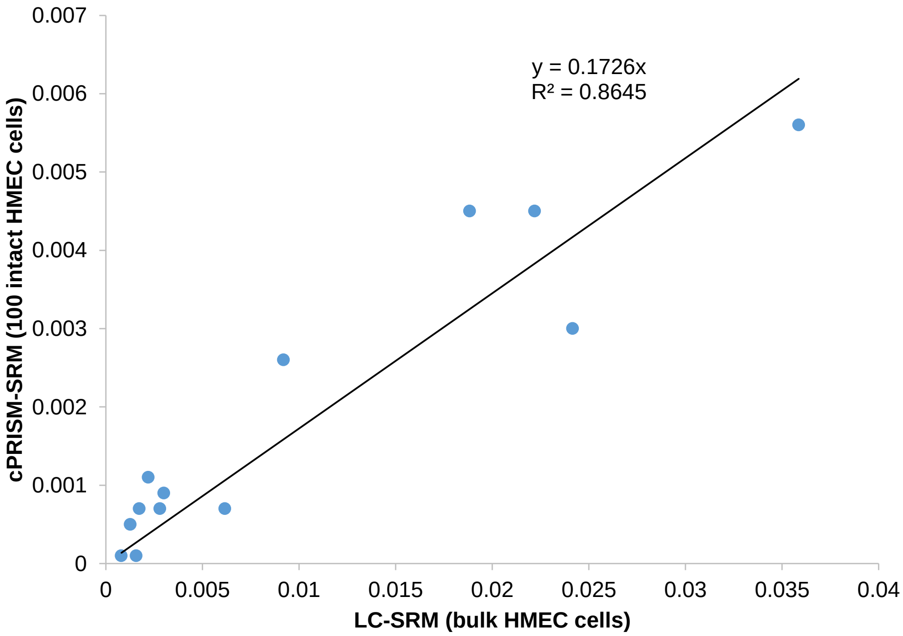

Supplementary Figure 15. Correlation analysis of targeted quantification of EGFR pathway proteins between 100 intact HMEC cells with cPRISM-SRM and bulk HMEC cells with LC-SRM to evaluate quantitation accuracy of cPRISM-SRM for a small number of cells.

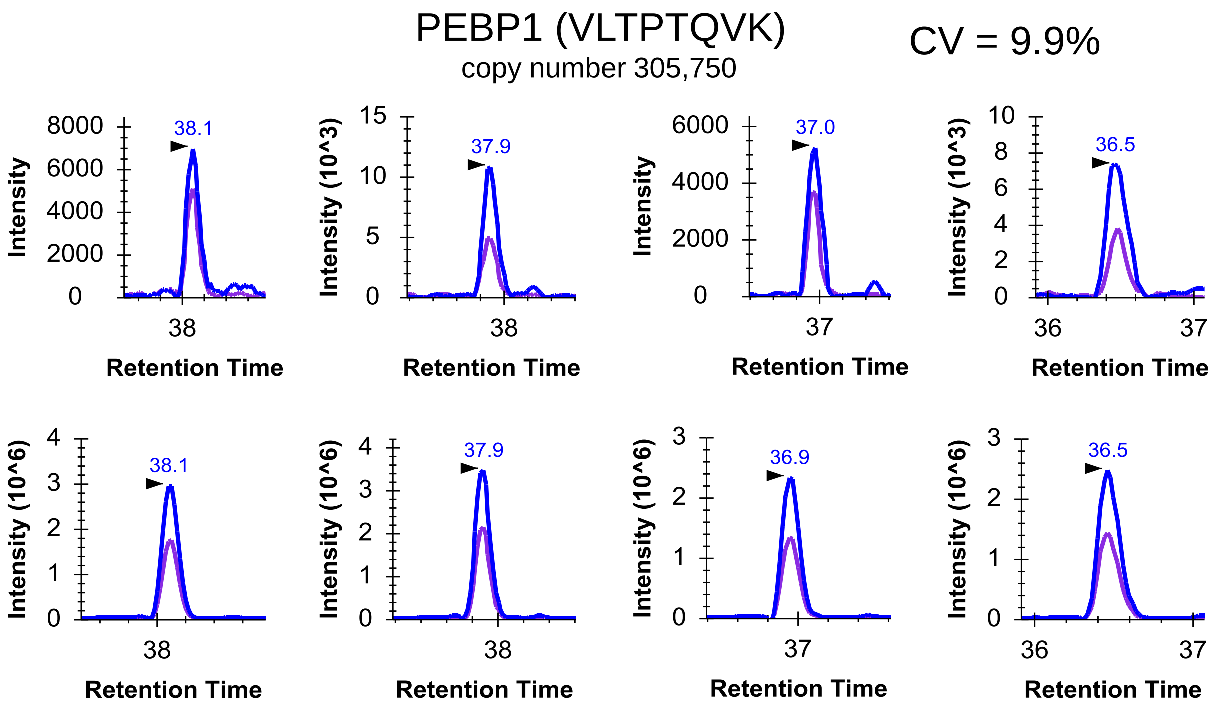

GRB2 (ATADDELSFK)

copy number 43,230

CV = 15.3%

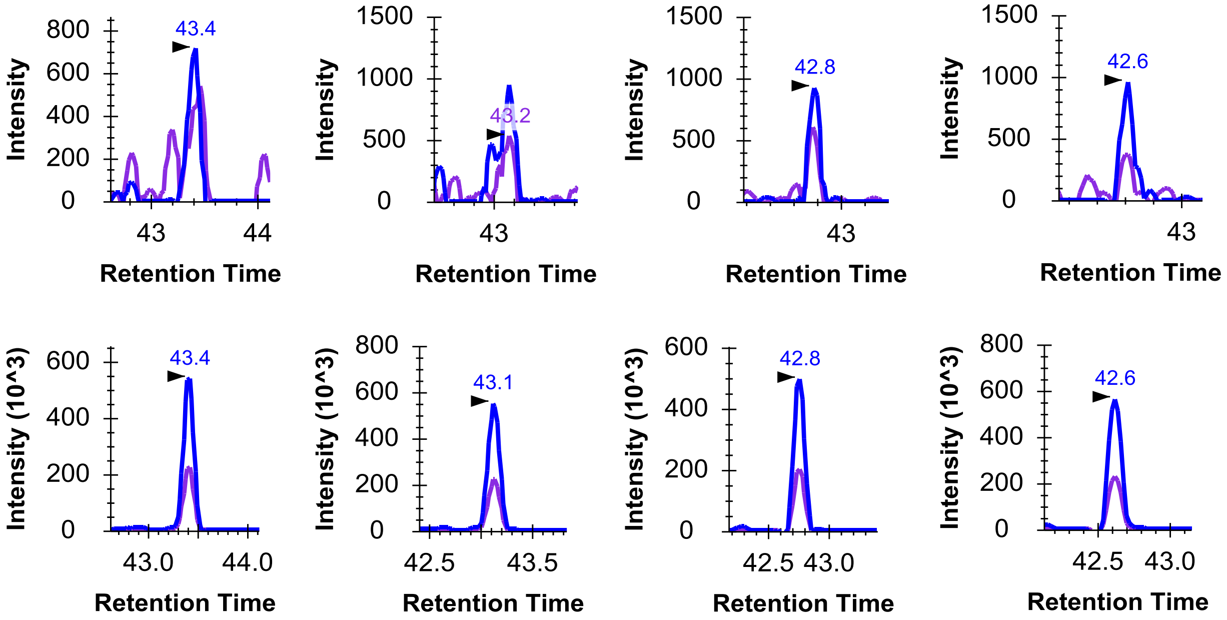

H/K/NRAS: LVVVGAGGVGK

copy number 213,232

CV = 8.9%

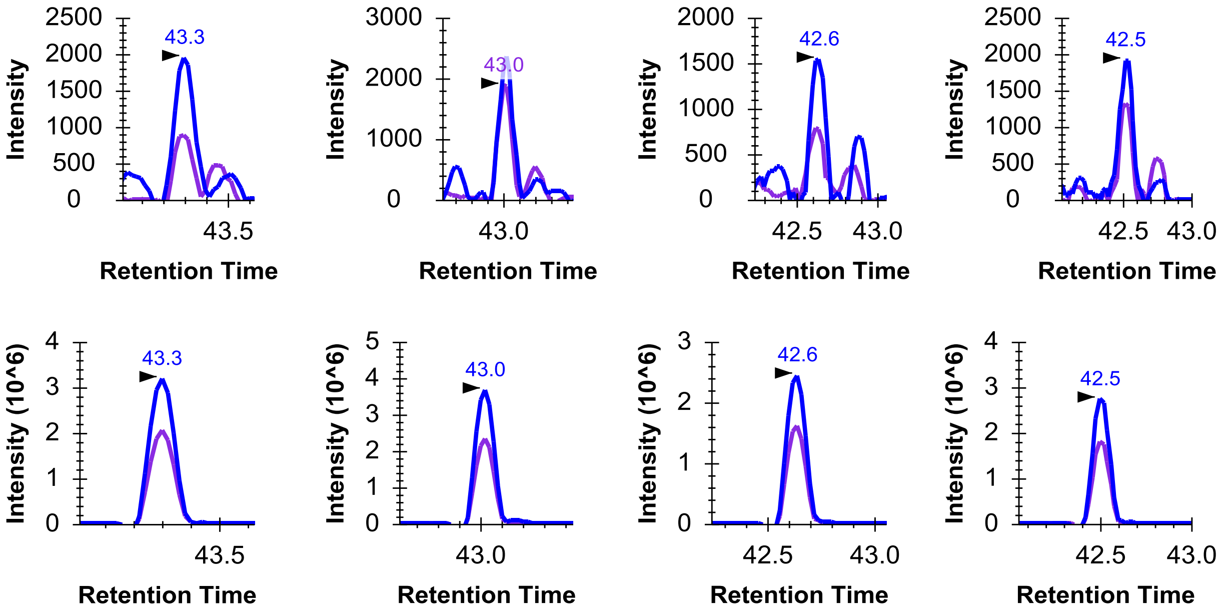

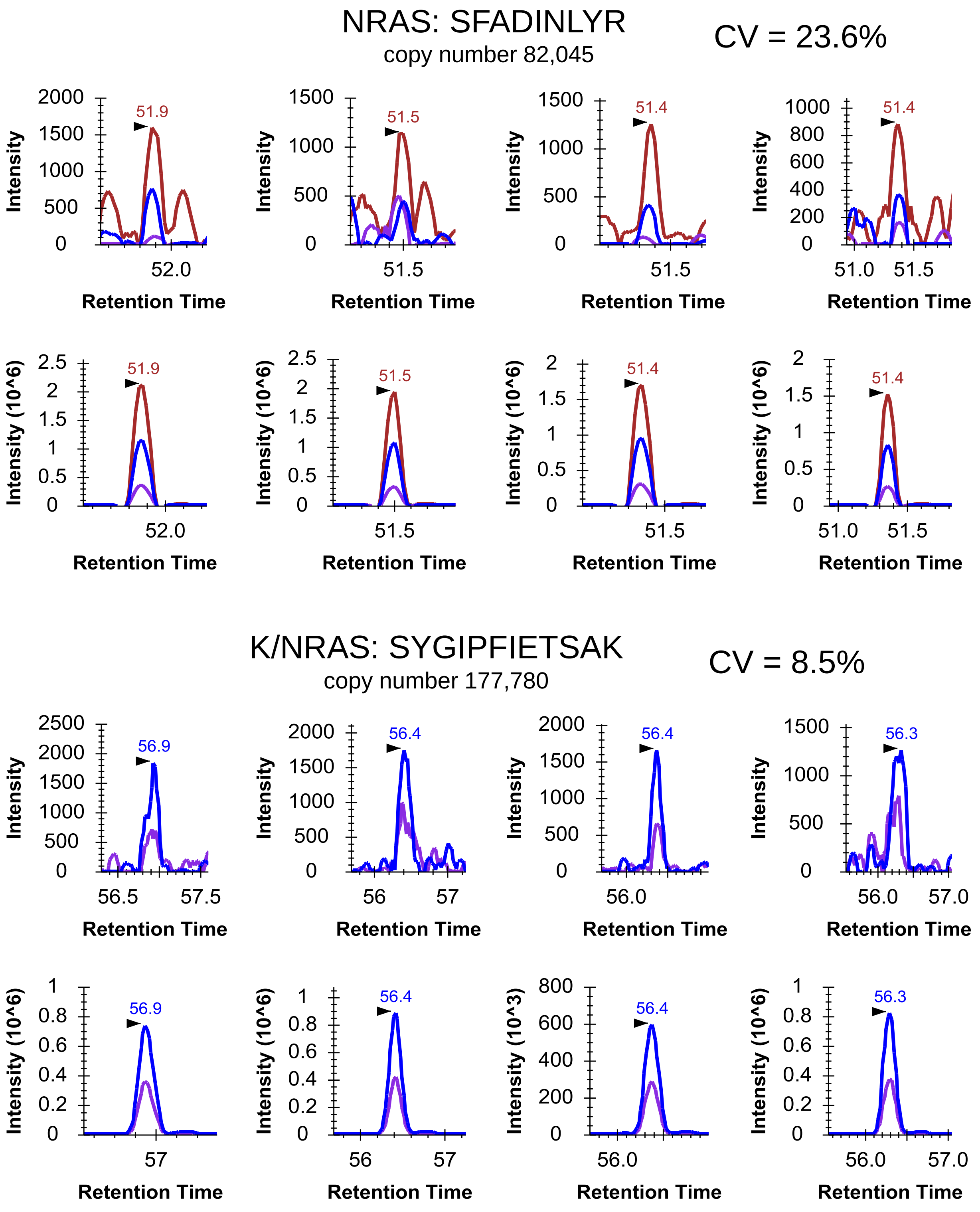

Supplementary Figure 16. Evaluation of the entire cPRISM-SRM reproducibility including sample preparation with four replicates for each target peptide at 100 intact HMEC cells.

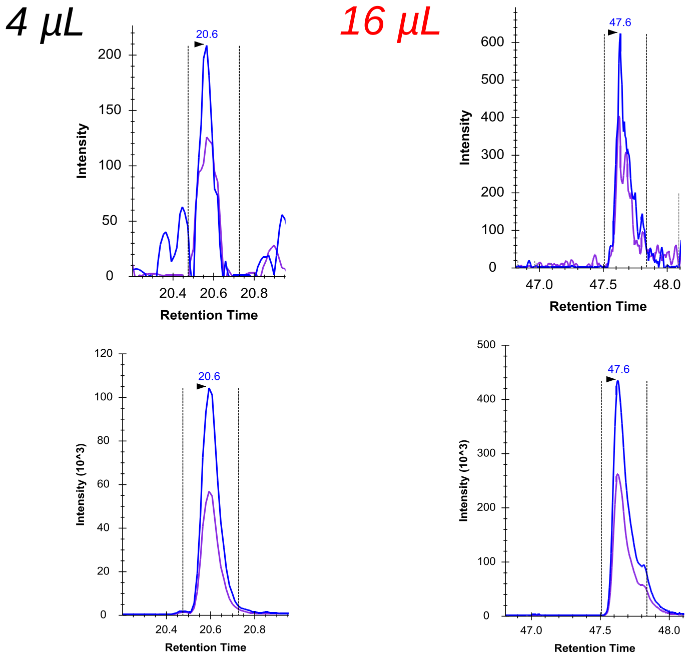

Supplementary Figure 17. An example of improved PRISM-SRM sensitivity at >3-fold by significantly increasing sample loading at the second dimensional LC separation from ~4 µL to ~16 µL with changing the loop from typical 5 µL to 20 µL. The total ~20 µL of PRISM fraction samples were nearly fully injected to maximize the detection sensitivity. The drawback is that there was no technical replicates for each PRISM fraction sample. As a consequence the loading time of the second dimensional LC separation was proportionally increased.

Supplementary Table 1. Absolute abundance (protein copies per cell) of EGFR pathway proteins in bulk HMEC cells from our previous study (Shi *et al.* Sci Signal 2016, 9:rs6). The surrogate peptides marked within red frames that represent high-, moderate-, and low-abundance were selected for cPRISM-SRM measurements across different equivalent numbers of HMEC cells (1-1000 cells) to evaluate its sensitivity.

Protein	Surrogate peptide	Protein copies per HMEC cell
ADAM17	NIYLNSGLTSTK	36080
AKT1	FYGAEIVSALDYLHSEK	1523
ARAF	TQHCDPEHFPFPAPANAPLQR	11661
CBL	GTEPIVVDPFDPR	11532
DUSP4	GSVSLEQILPAEEEVR	266
DUSP6	DSTNLDVLEEFGIK	164
EGFR	LTQLGTFEDHFLSLQR	354000
ERRFI1	NSPSLFPCAPLCER	5392
GAB1	LTGDPDVLEYYK	3332
GRB2	ATADDELSFK	43230
HRAS	SFEDIHQYR	68452
H/K/NRAS	LVVVGAGGVGK	213232
K/NRAS	SYGIPFIETSAK	177780
KRAS	SFEDIHHYR	95735
MAP2K1	ISELGAGNGGVVFK	22902
MAP2K1/2K2	LIHLEIKPAIR	63350
MAP2K2	ISELGAGNGGVVTK	44630
MAPK1	VADPDHDHTGFLTEYVATR	104400

Protein	Surrogate peptide	Protein copies per HMEC cell
MAPK3	IADPEHDHTGFLTEYVATR	67109
NRAS	SFADINLYR	82045
PDPK1	QLLLTEGPHLYYVDPVNK	19910
PEBP1	VLTPTQVK	305750
PIK3R1	FSAASSDNTENLIK	12372
PIK3R2	ALGATFGPLLLR	9839
PTPN11	SNPGDFTLSVR	25110
PTPRE	TGTFIALSNILER	13251
RAF1	TISNGFGFK	6754
RASA1	LLAITELLQQK	31197
SHC1	EAISLVCEAVPGAK	25055
SHOC2	LDSLTTLYLR	14429
SOS1	EYIQPVQLR	3671
SOS1	LPSADVYR	3094
SOS2	LPGYSSAEYR	1635
SPRED1	VPHQEENGCADFFIR	204
SPRY4	LQHPLTILPIDQVK	219

Supplementary Table 2. The SRM S/N ratio of endogenous peptides at 1-100 HMEC cell equivalents from bulk cell digests measured by cPRISM-SRM.

Protein	Surrogate peptide	Copy number per cell	S/N ratio						
			0 cell	1 cell	5 cell	10 cell	20 cell	50 cell	100 cell
EGFR	LTQLGTFEDHFLSLQR	354,000	n/a	n/a	n/a	n/a	7	12	41
H/K/NRAS	LVVVGAGGVGK	213,232	n/a	2	7	6	13	12	26
K/NRAS	SYGIPFIETSAK	177,780	4	8	8	14	9	12	15
NRAS	SFADINLYR	82,045	7	n/a	7	11	7	9	n/a
HRAS	SFEDIHQYR	68,452	n/a	n/a	n/a	n/a	2	5	7
ADAM17	NIYLNSGLTSTK	36,080	2	4	5	6	n/a	n/a	7
SHC1	EAISLVCEAVPGAK	25,055	n/a	3	5	8	n/a	9	7

*n/a: target peptide fractions were either not located or involved significant sample loss.

Supplementary Table 3. The cell line density (average number of cells per µg) (Shi *et al.* Sci Signal 2016, 9:rs6).

cell line	date	plate 1 - image 1	plate 1 - image 2	plate 2 - image 3	plate 2 - image 4	plate 1 - average	plate 2 - average	avg # cells/plate	total lysate volume (mL) - ELISA	lysate volume (mL)/plate	[lysate] (ug/mL)	avg protein (ug)/plate	# cells/ ug protein	# cells for 8ug protein	avg # cells/mg	avg # cells/µg
**BT-20**	10/6/2010	1.470	1.660	1.320	1.530	1.565	1.425	1.495E+07	9.0	1.286	2075.201	2668.116	5603.206	44825.64	5.603E+06	5.603E+03
**SKBR3**	11/18/2010	1.030	1.020	1.170	1.100	1.025	1.135	1.080E+07	10.0	1.429	1632.153	2331.647	4631.919	37055.35	4.632E+06	4.632E+03
**MCF-10A**	12/8/2010	1.540	1.460	1.370	1.330	1.500	1.350	1.425E+07	9.5	1.357	2010.587	2728.654	5222.355	41778.84	5.222E+06	5.222E+03
**MCF-7**	12/9/2010	0.930	0.850	0.800	0.810	0.890	0.805	8.475E+06	9.0	1.286	1311.595	1686.336	5025.688	40205.50	5.026E+06	5.026E+03
**MDA-MB-231**	2/2/2011	2.170	1.810	1.840	2.420	1.990	2.130	2.060E+07	9.0	1.286	2692.304	3461.534	5951.119	47608.95	5.951E+06	5.951E+03
**HMEC**															5.287E+06	5.287E+03
**NHDF**															5.287E+06	5.287E+03
**HS578T**															4.554E+06	4.554E+03

Based on the above information 1 µg of total protein is equivalent to ~5000 mammalian cells (i.e., ~200 pg per mammalian cell). Assuming 50% peptide recovery from protein digestion one mammalian cell can generate ~100 pg of total peptides.
