# Supplementary material for: Facile carrier-assisted targeted mass spectrometric approach for proteomic analysis of low numbers of mammalian cells
Source: Commun Biol. 2018 Aug 6;1:103. doi: 10.1038/s42003-018-0107-6 (PMC6123794; doi:10.1038/s42003-018-0107-6)
Supplement: Supplementary file 2 — Description of Supplementary Files [file 42003_2018_107_MOESM2_ESM.docx]

**Description of Additional Supplementary Files**

File Name: Supplementary Data 1

Description: MSGF+ searching results for LC-MS/MS analysis of BSA tryptic digest (searching both the human and bovine protein sequence database).

File Name: Supplementary Data 2

Description: Non-redundant unique BSA peptides from BSA tryptic digest.

File Name: Supplementary Data 3

Description: Using Thermo peptide analyzing tool to analyze BSA peptide hydrophobicity (high value and low value means that the peptide is very hydrophobic and very hydrophilic, respectively).

File Name: Supplementary Data 4

Description: Selected EGFR pathway proteins, their surrogate peptides, and the optimized transitions and collision energies.

File Name: Supplementary Data 5

Description: Selection of surrogate peptides measured by cPRISM-SRM for obtaining LODs and LOQs in 1-1000 HMEC cell equivalents.

File Name: Supplementary Data 6

Description: SRM quantification of EGFR pathway proteins in a panel of cell lines (please also see Shi et al. Sci Signal 2016, 9:rs6).

File Name: Supplementary Data 7

Description: Estimated EGFR pathway protein copy number in a panel of cell lines and the correlation between mRNA and protein expression levels across different cell lines (please also see Shi et al. Sci Signal 2016, 9:rs6).
